# Reflections on the use of the World Health Organization’s (WHO) OneHealth Tool: Implications for health planning in low and middle income countries (LMICs)

**DOI:** 10.12688/f1000research.13824.2

**Published:** 2018-03-05

**Authors:** John Q. Wong, Nel Jason Haw, Jhanna Uy, Diana Beatriz Bayani

**Affiliations:** 1 EpiMetrics Inc., Parañaque City, 1713, Philippines; 2 Health Policy Development and Planning Bureau, Department of Health, Manila, 1003, Philippines

**Keywords:** priority-setting, low and middle income countries, WHO CHOICE, generalized cost-effectiveness analysis, OneHealth Tool, sector-wide planning

## Abstract

The World Health Organization (WHO) launched the OneHealth Tool (OHT) to help low and middle income countries to develop their capacities for sector-wide priority setting. In 2016, we sought to use the OHT to aid the Philippine Health Insurance Corporation (PHIC), the national health insurer of the Philippines, in decisions to expand benefit packages using cost-effectiveness analyses. With technical support from the WHO, we convened health planning officers from the Philippine Department of Health (DOH) and the Philippine Health Insurance Corporation (PHIC) conduct generalized cost-effective analyses (GCEA) of selected un-financed noncommunicable disease interventions using OHT. We collected epidemiological and cost data through health facility surveys, review of literature such as cost libraries and clinical practice guidelines, and expert consultations.

Although we were unable to use GCEA results directly to set policy, we learnt important policy lessons which we outline here that might help inform other countries looking to inform service coverage decisions. Additionally, the entire process and GCEA visualizations helped high-level policymakers in the health sector, who have traditionally relied on ad hoc decision making, to realize the need for a systematic and transparent priority-setting process that can continuously provide the evidence needed to inform service coverage decisions.

## Introduction

Striving for universal health coverage requires priority setting to maximize health gains from limited resources ( Chalkidou *et al*., 2016). Countries like the Philippines struggle with building technical and institutional capacity for health technology assessment (HTA).

In 2015, the national insurer Philippine Health Insurance Corporation (PHIC) wanted to redefine their health benefits package. We developed a list of priority conditions based on disease burden and a corresponding list of cost-effective interventions ( Wong *et al*., 2018). This paper documents our subsequent attempt on a more definitive list of interventions using cost-effectiveness analyses (CEA) in the Philippine setting. At that time, however, we had insufficient technical capacity to conduct the volume of necessary primary CEAs within the timeframe of our policy window. We sought the help of the World Health Organization (WHO) to conduct generalized cost-effectiveness analysis (GCEA) using their OneHealth Tool (OHT) software.

The appeal of GCEA and OHT was its perceived feasibility for our purposes. OHT had been used successfully in 20 low and middle income countries (LMICs) for sector-wide planning ( Avenir Health, 2017), and WHO-CHOICE had conducted regional-level GCEA for a good range of diseases in our priority list of conditions ( World Health Organization, 2017). Because GCEA uses a “do nothing” comparator, we would be able to simultaneously analyze multiple diseases and new and existing alternatives within and outside the health sector to determine the most efficient mix of interventions to fund at the national-level ( Hutubessy *et al.*, 2003). Lastly, GCEA could be done through the user-friendly OHT software with its built-in health impact models, templates for costing and epidemiological inputs, and data visualization functions.

We hope that our reflections on our experience with GCEA using OHT will aid other LMICs looking to scale up their priority setting efforts.

## Methods

Based on the available models in the OHT at that time (April 2016), twelve interventions across three diseases - cardiovascular disease, diabetes, chronic obstructive pulmonary disorder - were chosen for analysis. OHT has global or regional defaults for all of the values in the models, and we attempted to replace all of the defaults with the best available local data. To do this, we collected epidemiological and cost data from health facility surveys, desk reviews, and clinical experts. A summary of data sources for each input is detailed in Table 1.

**Table 1.** Data sources used for inputs in the OneHealth Tool.

Input	Data source	Remarks
Composition of cost items under each intervention	Available local clinical practice guidelines (CPGs) Expert opinion	Global or United States CPGs were used if local CPGs were not available, as used by specialists in their clinical practice If no CPGs are utilized in the Philippine context, then opinions of the medical consultants in the workshop were sought
Demographic data	United Nations Population Division World Population Prospectus 2015	OHT lets users load country-specific demographic information upon set-up of a new project file; projections reflect Philippine census data in 2015
Disease epidemiology	Global Burden of Disease (GBD) 2013 data Modeling using DISMOD II 1.04 Desk review of published and gray literature	All inputs were replaced by a mix of these sources except the age-specific disability weights, as the defaults were retained as they were expected to be somewhat constant across different countries
Effectiveness of intervention	Desk review of published and gray literature	Some of the default data were retained when the effectiveness data came from a systematic review or meta-analysis
Coverage rates of intervention (target and expected)	Desk review of published and gray literature Interview with experts	Experts were recruited from various medical professional societies, and program managers from the DOH For items where more than one expert was consulted, the average was taken Some default data were retained when experts refused to estimate
Intervention costs	Primary data collection in 24 purposively selected health facilities across the Philippines (6 government hospitals, 9 government primary care clinics, 6 private hospitals, and 3 private clinics) 2015 Drug Price Reference Index (DPRI) Management Sciences for Health (MSH) International Drug Price Indicator Guide	The average of each cost item was taken across all facilities Overhead costs were estimated from available budgets of selected government hospitals Yearly list released by DOH on median procurement prices of certain drugs in government hospitals Used when drug price was not collected at the facility survey or not found in the DPRI
Program costs	DOH 2015 budget	

Note: A full documentation of the changes, together with the references and notes, are found in Supplementary File 1.

We invited WHO-Geneva to conduct a five-day workshop on the use of OHT, with DOH and PHIC planning officers, and some senior officials as participants. Over the course of the workshop, we used the default parameters of the model as a starting point of discussion. We used a consensus approach in modifying these defaults. We first consulted available data we had gathered, then all workshop participants discussed iteratively which data inputs and resource requirements were appropriate in the Philippine context until a consensus was reached.

**Figure 1.**
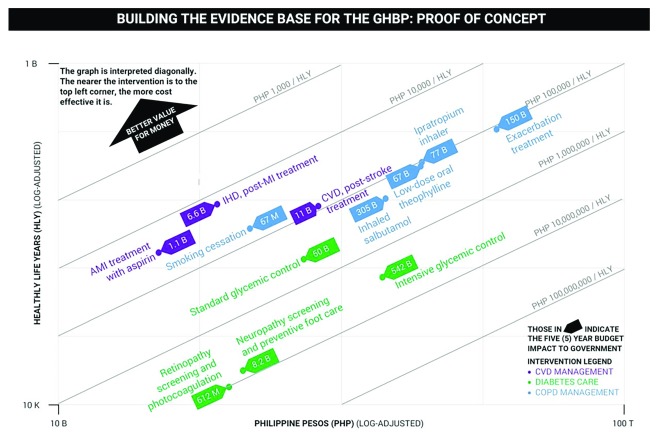
Isoquant graph of selected non-communicable interventions with budget impact price tags. Calculations made using OneHealth Tool.

On the last day, we plotted the cost-effectiveness results on an isoquant graph. Each intervention represents a point, and the graph is to be interpreted diagonally. The graph ( Figure 1) shows the cost of the intervention on the horizontal axis, and effectiveness on the vertical axis. We have also added price tags to each data point to indicate the five-year budget impact.

## Lessons learned

Our study raised awareness among policy makers that cost-effectiveness can be used for making decisions on service coverage. They found the GCEA results straightforward and understandable. However, for the policy question, GCEA methods were deemed insufficient to satisfy policy makers’ demands. GCEA was geared for choosing a complement of mutually compatible interventions based on a ‘do-nothing’ scenario. The results ended up validating the programs already covered, but failed to address the question of expanding service coverage. We realized that a CEA with current practice as the comparator would be more appropriate.

Another limitation of GCEA is the limited availability of models, making it difficult to conduct a sector-wide exercise that completely incorporates the Philippine baseline context. While more models have been added since our last use, such as modules on neglected tropical diseases, it will take time before the full breadth of interventions will be covered. Most of the disease packages available were based on WHO’s list of global priorities, which were already covered in the Philippines. In addition, the process of modifying defaults in the OHT was more difficult than expected. Expert opinion and clinical practice guidelines varied widely, which made it challenging to reconcile the input values, especially in the absence of a local methods guideline for economic evaluation.

The undertaking also led to the policy makers’ realization that no systematic process existed within PHIC for developing benefit packages. The failure in providing an answer to the question of “Which intervention to cover next?” through the GCEA pushed all stakeholders to consider setting up a process and value framework for priority setting alongside an institution that will facilitate such processes. While PHIC and DOH are not completely new to HTA, there was a need to establish a single, harmonized priority setting process for the health sector that would enable these two institutions to make better policy decisions based the questions they are currently faced with. This envisioned an HTA system, characterized by societal principles, with specific criteria and steps and supporting legal framework that would respond to the next level of questions such as which drug to reimburse, given that both institutions are flooded by demands from various stakeholders to cover a wide range of interventions.

This whole exercise re-emphasizes that defining a health benefits package cannot be a one-off exercise. It is influenced by a myriad of factors, and entails much stakeholder consultation and awareness of societal and institutional values. We recognize that the next step for the Philippines is to embed in the health system a process of both evidence generation and use for making investment decisions in the public sector. Developing capacity at both technical and sectoral dimensions must also be prioritized. Despite the limitations of the GCEA, it was a practical exercise to demonstrate the importance of evidence use in supporting policy surrounding service coverage decisions.

## Data and software availability

The OneHealth Tool (OHT) may be downloaded freely at from Avenir Health. At the time this study was conducted, the Spectrum version of the OHT was 5.42. The results may be recreated by following the detailed documentation of changes to the OHT defaults found in Supplementary File 1.

Data entered into OHT was obtained from sources listed in Table 1

## Supplementary material

**Supplemental File 1. Documentation of changes to WHO OneHealth Tool (OHT).** Input-by-input changes to the interventions considered to recreate the results of the study.
