# Supplementary material for: Reflections on the use of the World Health Organization’s (WHO) OneHealth Tool: Implications for health planning in low and middle income countries (LMICs)
Source: F1000Res. 2018 Mar 5;7:157. Originally published 2018 Feb 7. [Version 2] doi: 10.12688/f1000research.13824.2 (PMC5832917; doi:10.12688/f1000research.13824.2)
Supplement: Supplementary file 1 [file f1000research-7-15444-s0000.tgz › e64684fc-9175-4530-b970-4f20d468ce3c.docx]

**Supplementary File 1**

Documentation of changes to WHO OneHealth Tool (OHT)

**NON-COMMUNICABLE DISEASES**

Prepared by:

Haw, Nel Jason

Uy, Jhanna

Bayani, Diana Beatriz

Wong, John

Version as of June 2016

**TABLE OF CONTENTS**

[**1. Chronic Obstructive Pulmonary Disorder (COPD)**](#_wce4ykg9mo9) **3**

[EPIDEMIOLOGY](#_v42n1ade8rj1) 3

[DISMOD II Inputs](#_3yve5eciw2de) 3

[Health State: Disease free/Susceptible to Transition: Incidence](#_e03i5mpy3mw8) 5

[Health State: COPD to Transition: Case-fatality rate](#_2myeobw4x0hp) 6

[Health State: COPD to Transition: Remission](#_6j9bp0u8w3nt) 6

[INTERVENTION COSTING](#_wel8bon5mcdv) 7

[Target Population](#_2ffz49im4hzw) 7

[Population-in-Need](#_y3prurellamd) 7

[Intervention Coverage](#_tjfn466oluyr) 8

[Treatment Inputs](#_dqf5iqs6u3qs) 9

[Delivery Channels](#_gnsyj6kl6d0b) 15

[**2. Diabetes**](#_31y61vheblnz) **16**

[EPIDEMIOLOGY](#_4h9e1cdoecdt) 16

[DISMOD II Inputs](#_64f9ghc9efu) 16

[Health State: Disease free/Susceptible to Transition: Incidence](#_fctk3hb93892) 19

[Health State: Diabetes to Transition: Incidence](#_jm2clypqtlft) 20

[Health State: Diabetes to Transition: Case-fatality rate](#_cr6t868y6gcj) 21

[Health State: Diabetic Blindness to Transition: Case-fatality rate](#_aug55z9kelnz) 22

[Health State: Diabetic Lower Extremity Amputation to Transition: Case-fatality rate](#_l3xp1iko4g0m) 23

[INTERVENTION COSTING](#_aw3jvcqthjvk) 24

[Target Population](#_rooyhzdjjywd) 24

[Population-in-Need](#_8uvps61afw8o) 24

[Intervention Coverage](#_x84auhm96jtz) 24

[Treatment Inputs](#_z3s4yxc5xanj) 25

[Delivery Channels](#_tun031f2w71i) 32

[**3. CVD/Diabetes Screening, CVD Management**](#_11yxt89ulmrm) **33**

[EPIDEMIOLOGY](#_mq8u8qqid33p) 33

[INTERVENTION COSTING](#_wtdb9aa7tuqc) 33

[Target Population](#_5wctsz7llhad) 33

[Population-in-Need](#_utsbxtbg4mcy) 33

[Intervention Coverage](#_b27z591r5byb) 33

[Treatment Inputs](#_o4ldbvdetuyh) 34

[Delivery Channels](#_y2i7q7coq4tw) 44

[**4. and 5. MATERNAL AND CHILD**](#_3809zuetivor) **46**

[LiST Impact Module Epidemiology](#_fe7b3ffvlokc) 46

[INTERVENTION COSTING](#_qkkquzo9lby3) 52

[Target Population](#_mkomcq78rupx) 52

[Population-in-Need](#_ngkm985p70bm) 52

[Intervention Coverage](#_iwcw1cy0e3wc) 54

[Treatment Inputs](#_atqird9dx8ov) 55

[Outpatient and Inpatient Costs](#_e0b4afhfiet5) 69

[Delivery Channels](#_i5st1ic0yhff) 70

[Price Comparison between Default and Replaced](#_lz43rwr8t88i) 72

[GCEA RESULTS](#_r39gm7aatein) 73

# 1. Chronic Obstructive Pulmonary Disorder (COPD)

## EPIDEMIOLOGY

### DISMOD II Inputs

Associated health states were modeled from DISMOD II using the following inputs:

Table 1.1. COPD inputs for DISMOD II

| Age group | Prevalence (per 1,000) | | Mortality (per 100,000) | | Remission |
| --- | --- | --- | --- | --- | --- |
|  | From 2010 Global Burden of Disease data on Philippines | | From the General Record of Incidence of Mortality (GRIM), Australia 2013 | | COPD patients do not recover from COPD |
|  | Male | Female | Male | Female | Male/Female |
| 0 to 4 | 2.992991 | 1.780535 | 0.1 | 0.1 | 0 |
| 5 to 9 | 13.04122 | 9.034464 | 0 | 0 | 0 |
| 10 to 14 | 23.58253 | 17.0458 | 0 | 0 | 0 |
| 15 to 19 | 35.09937 | 25.42391 | 0 | 0.1 | 0 |
| 20 to 24 | 50.33278 | 37.55522 | 0 | 0 | 0 |
| 25 to 29 | 63.35752 | 48.08461 | 0 | 0.1 | 0 |
| 30 to 34 | 73.52938 | 58.15146 | 0 | 0 | 0 |
| 35 to 39 | 84.47309 | 69.04364 | 0.3 | 0.1 | 0 |
| 40 to 44 | 96.92152 | 81.63986 | 1.1 | 0.1 | 0 |
| 45 to 49 | 119.1284 | 103.6187 | 1.7 | 1.6 | 0 |
| 50 to 54 | 146.5107 | 130.9996 | 3.9 | 4.1 | 0 |
| 55 to 59 | 188.2787 | 174.1115 | 11.8 | 10.2 | 0 |
| 60 to 64 | 199.8073 | 175.3001 | 26.7 | 18.7 | 0 |
| 65 to 69 | 252.1461 | 228.8193 | 58.3 | 41.5 | 0 |
| 70 to 74 | 253.4048 | 237.7502 | 121.3 | 81.1 | 0 |
| 75 to 79 | 287.2998 | 270.8643 | 215.2 | 139.2 | 0 |
| 80 to 84 | 303.8194 | 328.6827 | 367.2 | 235.4 | 0 |
| 85 above |  |  | 762.2 | 380 | 0 |

Prevalence statistics were provided by WHO.

Mortality rates available from the following [URL](https://www.aihw.gov.au/reports/life-expectancy-death/grim-books/contents/grim-books).

###

### Health State: Disease free/Susceptible to Transition: Incidence

Table 1.2. **Male** COPD Inputs for GCEA. Those in gray mean that defaults were accepted.

| Age group | Disability weight | Prevalence  (per 1,000) | Transitions - Incidence | |
| --- | --- | --- | --- | --- |
|  |  |  | Rates (per 1,000) | To health state |
| 0 to 4 | 0.03 | Grayed out (Prevalence rate changed in “Health State: COPD episode”) | 0.4125 | COPD episode |
| 5 to 9 | 0.05 |  | 2.3096 | COPD episode |
| 10 to 14 | 0.06 |  | 2.4899 | COPD episode |
| 15 to 19 | 0.07 |  | 2.3727 | COPD episode |
| 20 to 24 | 0.09 |  | 3.1646 | COPD episode |
| 25 to 29 | 0.10 |  | 2.3633 | COPD episode |
| 30 to 39 | 0.12 |  | 3.0269 | COPD episode |
| 40 to 49 | 0.15 |  | 5.3037 | COPD episode |
| 50 to 59 | 0.18 |  | 9.9639 | COPD episode |
| 60 to 69 | 0.21 |  | 5.7124 | COPD episode |
| 70 to 79 | 0.23 |  | 9.5113 | COPD episode |
| 80 to 100 | 0.27 |  | 7.6916 | COPD episode |

Table 1.3. **Female** COPD Inputs for GCEA. Those in gray mean that defaults were accepted.

| Age group | Disability weight | Prevalence  (per 1,000) | Transitions - Incidence | |
| --- | --- | --- | --- | --- |
|  |  |  | Rates (per 1,000) | To health state |
| 0 to 4 | 0.03 | Grayed out (Prevalence rate changed in “Health State: COPD episode”) | 0.2925 | COPD episode |
| 5 to 9 | 0.04 |  | 1.6872 | COPD episode |
| 10 to 14 | 0.06 |  | 1.8322 | COPD episode |
| 15 to 19 | 0.07 |  | 1.808 | COPD episode |
| 20 to 24 | 0.09 |  | 2.4117 | COPD episode |
| 25 to 29 | 0.10 |  | 1.9605 | COPD episode |
| 30 to 39 | 0.12 |  | 2.4957 | COPD episode |
| 40 to 49 | 0.15 |  | 5.5406 | COPD episode |
| 50 to 59 | 0.18 |  | 9.1825 | COPD episode |
| 60 to 69 | 0.20 |  | 5.5911 | COPD episode |
| 70 to 79 | 0.23 |  | 11.29 | COPD episode |
| 80 to 100 | 0.27 |  | 9.3937 | COPD episode |

### Health State: COPD to Transition: Case-fatality rate

Table 1.4. **Male** COPD Inputs for GCEA. Those in gray mean that defaults were accepted.

| Age group | Disability weight | Prevalence  (per 1,000) | Transitions - Case-fatality rate | |
| --- | --- | --- | --- | --- |
|  |  |  | Rates (per 1,000) | To health state |
| 0 to 4 | 0.15 | 3.5449 | 0.2853 | Deceased |
| 5 to 9 | 0.15 | 10.0084 | 0.1069 | Deceased |
| 10 to 14 | 0.15 | 23.1448 | 0.011 | Deceased |
| 15 to 19 | 0.15 | 34.1362 | 0.007 | Deceased |
| 20 to 24 | 0.15 | 47.9774 | 0.006 | Deceased |
| 25 to 29 | 0.15 | 61.0399 | 0.0117 | Deceased |
| 30 to 39 | 0.15 | 78.5045 | 0.0353 | Deceased |
| 40 to 49 | 0.15 | 114.1131 | 0.1058 | Deceased |
| 50 to 59 | 0.15 | 180.615 | 0.3478 | Deceased |
| 60 to 69 | 0.15 | 239.5371 | 1.6048 | Deceased |
| 70 to 79 | 0.15 | 289.8815 | 4.7673 | Deceased |
| 80 to 100 | 0.15 | 332.4832 | 12.9701 | Deceased |

Table 1.5. **Female** COPD Inputs for GCEA. Those in gray mean that defaults were accepted.

| Age group | Disability weight | Prevalence  (per 1,000) | Transitions - Case-fatality rate | |
| --- | --- | --- | --- | --- |
|  |  |  | Rates (per 1,000) | To health state |
| 0 to 4 | 0.14 | 2.1594 | 0.4797 | Deceased |
| 5 to 9 | 0.14 | 6.8641 | 0.178 | Deceased |
| 10 to 14 | 0.14 | 16.5302 | 0.0342 | Deceased |
| 15 to 19 | 0.14 | 24.8302 | 0.0409 | Deceased |
| 20 to 24 | 0.14 | 35.4839 | 0.033 | Deceased |
| 25 to 29 | 0.14 | 45.7909 | 0.0228 | Deceased |
| 30 to 39 | 0.14 | 61.5974 | 0.0154 | Deceased |
| 40 to 49 | 0.14 | 93.7532 | 0.049 | Deceased |
| 50 to 59 | 0.14 | 162.6503 | 0.347 | Deceased |
| 60 to 69 | 0.14 | 217.69 | 1.2867 | Deceased |
| 70 to 79 | 0.14 | 274.7202 | 3.4575 | Deceased |
| 80 to 100 | 0.14 | 347.9293 | 7.5726 | Deceased |

### Health State: COPD to Transition: Remission

Remission assumed to be zero as patients do not recover from COPD.

## INTERVENTION COSTING

### Target Population

All interventions address people with COPD - defaults retained

### Population-in-Need

Across all years:

Table 1.6. Population-in-need inputs in GCEA.

| Intervention | Default | Changed | Rationale / Sources |
| --- | --- | --- | --- |
| COPD: Smoking cessation | 67.1% | 38.61% | Based on a study by [Idolor, et al. (2011)](https://www.ncbi.nlm.nih.gov/pubmed/21801277) in two rural municipalities in Nueva Ecija  COPD prevalence: 20.8%  Sample size: 772  Number of current smokers with COPD: 62  % of COPD patients who smoke 62 / (772 * 20.8%) = 38.61% |
| COPD: Inhaled salbutamol  (representing SABA) | 100.0% | 100.0% | According to the Philippine College of Chest Physicians (PCCP) CPG on COPD Management:  “Recommended first-line brochodilators are beta 2-agonists and or anticholinergic agents.” Salbutamol is a beta 2-agonist.  All patients assumed to require SABA as it is first-line drug |
| COPD: Low-dose oral theophylline  **RENAMED AS**  COPD: Add-on Long-acting Bronchodilators for Moderate COPD | 44.0% | 47% | According to PCCP CPG: “Methylxanthine derivatives are recommended only as second-line bronchodilators.”  Thus, theophylline was removed and renamed into long-acting bronchodilators. PCCP CPG recommends that this be given to COPD patients with severity of moderate and above. PCCP CPG reports that the patient mix of mild to moderate above COPD is 53%:47% |
| COPD: Ipratropium inhaler  (representing SAMA) | 15.4% | 100% | See comment under “COPD: Inhaled salbutamol” since ipratropium is an anticholinergic agent  All patients assumed to require SAMA as it is first-line drug |
| COPD: Exacerbation treatment with antibiotics | 12.0% | 63% | Based from a prospective cohort study by [Garcia-Aymerich, et al. (2003)](https://www.ncbi.nlm.nih.gov/pubmed/12554887) following up 340 patients with COPD in Barcelona, Spain over a mean period of 1.1 years. 63% represent those with at least one readmission episode during the follow-up period.  Study was cited in the Philippine College of Chest Physicians (PCCP) CPG on COPD Management, 2009 as Philippine-based rates of ECOPD were not available. PIN % the same across all groups as all three interventions are packaged as one exacerbation treatment package.  PCCP CPG available upon request from the PCCP - not available online |
| COPD: Exacerbation treatment with oral prednisolone | 12.0% | 63% |  |
| COPD: Exacerbation treatment with oxygen | 12.0% | 63% |  |

###

### Intervention Coverage

This pertains to the “Baseline Coverage from impact module (2016)” column:

Table 1.7. Intervention coverage inputs in GCEA

| Intervention | Default | Changed | Rationale / Sources |
| --- | --- | --- | --- |
| COPD: Smoking cessation | 33% | 10.19% | Expert survey result (weighted according to certainty of the expert’s estimate) |
| COPD: Inhaled salbutamol | 33% | 55.00% |  |
| COPD: Add-on Long-acting Bronchidilators for Moderate COPD | 33% | 58.91% |  |
| COPD: Ipratropium inhaler | 33% | 29.44% |  |
| COPD: Exacerbation treatment with antibiotics | 33% | 15.63% |  |
| COPD: Exacerbation treatment with oral prednisolone | 33% | 50.63% |  |
| COPD: Exacerbation treatment with oxygen | 33% | 39.41% |  |

###

###

### Treatment Inputs

Table 1.8. Treatment inputs for **COPD: Smoking Cessation** in GCEA

| **Intervention Name: COPD: Smoking Cessation** | | | | | | | | |
| --- | --- | --- | --- | --- | --- | --- | --- | --- |
| DRUGS AND SUPPLIES | | | | | | | | |
| Drug/supply | % rec asp of treat | Note | # units | Times per day | Days per case | Units per case | Unit cost (PHP) 2015 | Cost per ave case (PHP) 2015 |
|  |  | PCCP CPG only recommends consultation as adequate intervention, but smoking cessation will also include the required spirometry test |  |  |  |  |  |  |
| MEDICAL PERSONNEL | | | | | | | | |
| Staff type | % | Note | Minutes | | # of days/visits | | | Total |
| Generalist | 100 | Changed time to 3 minutes as per PCCP CPG for smoking cessation | 3 | | 1 | | | 3 |
| OUTPATIENT VISITS AND INPATIENT DAYS | | | | | | | | |
| Type | % | Note | Units per case | | | | | Total |
| Outpatient visit | 100 | Default | 1 | | | | | 1 |

Table 1.9. Treatment inputs for **COPD: Inhaled salbutamol** in GCEA

| **Intervention Name: COPD: Inhaled salbutamol** | | | | | | | | |
| --- | --- | --- | --- | --- | --- | --- | --- | --- |
| DRUGS AND SUPPLIES | | | | | | | | |
| Drug/supply | % rec asp of treat | Note | # units | Times per day | Days per case | Units per case | Unit cost (PHP) 2015 | Cost per ave case (PHP) 2015 |
| Salbutamol inhaler, 100 mcg | 100 | Changed price (69.92 DPRI price + 30% markup = 90.896, assumed 200 doses = 0.45448; 1 puff every 4 hours as per PCCP CPG | 1.0 | 6 | 365 | 2,190 | 0.45 | 986.49 |
| Spirometry | 100 | The presence of post-bronchodilator FEV1/FVC <0.70 confirms the presence of persistent airflow limitation and thus of COPD (GOLD Report 2014)  Changed price according to lowest price found online | 1 | 1 | 1 | 1 | 1,000 | 1,000.00 |
| MEDICAL PERSONNEL | | | | | | | | |
| Staff type | % | Note | Minutes | | # of days/visits | | | Total |
| Generalist | 100 | No specific guideline in PCCP CPG; estimates made by team MDs | 10 | | 12 | | | 120 |
| Generalist | 100 | Added 22 minutes for spirometry: You have to wait 15 minutes after giving SABA to perform the exam. Estimate the actual exam to be 7 minutes. | 22 | | 1 | | | 22 |
| Nurse | 100 | Removed | 0 | | 0 | | | 0 |
| OUTPATIENT VISITS AND INPATIENT DAYS | | | | | | | | |
| Type | % | Note | Units per case | | | | | Total |
| Outpatient visit | 100 | No specific guideline in PCCP CPG; estimates made by team MDs | 12 | | | | | 12 |

Table 1.10. Treatment inputs for **COPD: Add-on Long-acting Bronchodilators for Moderate COPD** in GCEA

| **Intervention Name: COPD: Add-on Long-acting Bronchodilators for Moderate COPD** | | | | | | | | |
| --- | --- | --- | --- | --- | --- | --- | --- | --- |
| DRUGS AND SUPPLIES | | | | | | | | |
| Drug/supply | % rec asp of treat | Note | # units | Times per day | Days per case | Units per case | Unit cost (PHP) 2015 | Cost per ave case (PHP) 2015 |
| Tiotropium, inhaled caps, 18g/cap, 30 caps + inhaler | 50% | PCCP CPG recommends 1 cap daily; represents LAMA as it is most commonly prescribed, assumption of equal likelihood of prescription with LABA  Inhaler cost according to MIMS: 263, 30 tabs: 2531 | 1 | 1 | 365 | 365 | 83.93 | 30,365 |
| Formoterol fumarate, inhaled caps, 12g/cap | 50% | PCCP CPG recommends 1-2 cap daily (median: 1.5); represents LABA as it is most commonly prescribed; assumption of equal likelihood of prescription with LAMA  Inhaler cost according to MIMS: 263, 30 tabs: 984 | 1.5 | 1 | 365 | 547.5 | 33.07 | 12,071 |
| MEDICAL PERSONNEL | | | | | | | | |
| Staff type | % | Note | Minutes | | # of days/visits | | | Total |
| Generalist | 100 | See comment in Table 1.9 | 10 | | 12 | | | 120 |
| OUTPATIENT VISITS AND INPATIENT DAYS | | | | | | | | |
| Type | % | Note | Units per case | | | | | Total |
| Outpatient visit | 100 | See comment in Table 1.9 | 12 | | | | | 12 |

Price data from MIMS Philippines (mims.com.ph). Sign-in required.

Table 1.11. Treatment inputs for **COPD: Ipratropium inhaler** in GCEA

| **Intervention Name: COPD: Ipratropium inhaler** | | | | | | | | |
| --- | --- | --- | --- | --- | --- | --- | --- | --- |
| DRUGS AND SUPPLIES | | | | | | | | |
| Drug/supply | % rec asp of treat | Note | # units | Times per day | Days per case | Units per case | Unit cost (PHP) 2015 | Cost per ave case (PHP) 2015 |
| Ipratropium bromide 20 mcg | 100 | Changed price: MSH median price: 0.328 USD (2014), 2014 average USD:PHP exchange rate of 44.39; 2 puffs QID as prescribed in the PCCP CPG | 1.0 | 8 | 365 | 2,920 | 1.46 | 4,263.20 |
| MEDICAL PERSONNEL | | | | | | | | |
| Staff type | % | Note | Minutes | | # of days/visits | | | Total |
| Generalist | 100 | See comment in Table 1.9 | 10 | | 12 | | | 120 |
| OUTPATIENT VISITS AND INPATIENT DAYS | | | | | | | | |
| Type | % | Note | Units per case | | | | | Total |
| Outpatient visit | 100 | See comment in Table 1.9 | 12 | | | | | 12 |

Table 1.12. Treatment inputs for **COPD: Exacerbation treatment with antibiotics** in GCEA

| **Intervention Name: COPD: Exacerbation treatment with antibiotics** | | | | | | | | |
| --- | --- | --- | --- | --- | --- | --- | --- | --- |
| DRUGS AND SUPPLIES | | | | | | | | |
| Drug/supply | % rec asp of treat | Note | # units | Times per day | Days per case | Units per case | Unit cost (PHP) 2015 | Cost per ave case (PHP) 2015 |
| Amoxicillin, 500 mg tab | 56% | According to PCCP CPG guidelines, 80% of exacerbations are infectious in origin, 70% of which are bacterial  Amoxicillin used as “tracer” antibiotic, representing first-line treatment for Group A COPD, assumption of 3x a day. PCCP CPG recommends 5 day therapy adequate for COPD treatment  Changed price (1.26 DPRI price + 30% markup = 1.64) | 1.0 | 3 | 5 | 15 | 1.64 | 24.60 |
| Chest radiograph | 100 | Useful in excluding other diagnoses (GOLD 2014)  Price taken from lowest cost in costing survey | 1 | 1 | 1 | 1 | 125 | 125 |
| Complete Blood Count | 100 | It may identify polycythemia (hematocrit >55%), anemia, or leukocytosis (GOLD 2014)  Price taken from lowest cost in costing survey | 1 | 1 | 1 | 1 | 70 | 70 |
| Sputum culture and antibiotic sensitivity | 100 | The presence of purulent sputum during an exacerbation can be sufficient indication for starting empirical antibiotic treatment.  Price taken from lowest cost in costing survey | 1 | 1 | 1 | 1 | 1,000 | 1,000 |
| MEDICAL PERSONNEL | | | | | | | | |
| Staff type | % | Note | Minutes | | # of days/visits | | | Total |
| Generalist | 100 | No specific guideline in PCCP CPG; estimates made by team MDs  Nurse is expected to deliver antibiotics to patient 3 times a day, while doctor sees patient during the first and last day of inpatient visit | 20 | | 2 | | | 40 |
| Nurse | 100 |  | 10 | | 15 | | | 150 |
| Laboratory technicians/ assistants | 100 | To conduct the test | 10 | | 1 | | | 10 |
| Radiographers/X-ray technicians | 100 | To conduct the test | 15 | | 1 | | | 15 |
| OUTPATIENT VISITS AND INPATIENT DAYS | | | | | | | | |
| Type | % | Note | Units per case | | | | |  |
| Inpatient days | 100 | PCCP CPG recommends 5 day stay as adequate | 5 | | | | | 5 |

Table 1.13. Treatment inputs for **COPD: Exacerbation treatment with oral prednisolone** in GCEA

| **Intervention Name: COPD: Exacerbation treatment with oral prednisolone** | | | | | | | | |
| --- | --- | --- | --- | --- | --- | --- | --- | --- |
| DRUGS AND SUPPLIES | | | | | | | | |
| Drug/supply | % rec asp of treat | Note | # units | Times per day | Days per case | Units per case | Unit cost (PHP) 2015 | Cost per ave case (PHP) 2015 |
| Prednisolone, tablet, 20 mg | 63.5% | Garcia-Aymerich, et al. (2003) reports that 40% of the cohort had two or more readmissions during the follow-up period, this represents 63.5% of all readmissions. PCCP CPG recommend that oral prednisolone be only given for repeated exacerbations. This is approximated as having more than one exacerbation episode during the follow-up period of the Garcia-Aymerich study.  40 mg for 5 days recommended according to [GOLD 2014](https://www.philhealth.gov.ph/circulars/2015/circ007-2015.pdf)  Changed price (4.50 DPRI price + 30% markup = 5.85) | 2.0 | 1 | 5 | 10 | 5.85 | 37.15 |
| MEDICAL PERSONNEL | | | | | | | | |
| Staff type | % | Note | Minutes | | # of days/visits | | | Total |
| Generalist | 100 | See comment in Table 1.12 | 20 | | 2 | | | 40 |
| Nurse | 100 |  | 10 | | 5 | | | 50 |
| OUTPATIENT VISITS AND INPATIENT DAYS | | | | | | | | |
| Type | % | Note | Units per case | | | | |  |
| Outpatient visit | 100 | See comment in Table 1.12 | 5 | | | | | 5 |

Table 1.14. Treatment inputs for **COPD: Exacerbation treatment with oxygen** in GCEA

| **Intervention Name: COPD: Exacerbation treatment with oxygen** | | | | | | | | |
| --- | --- | --- | --- | --- | --- | --- | --- | --- |
| DRUGS AND SUPPLIES | | | | | | | | |
| Drug/supply | % rec asp of treat | Note | # units | Times per day | Days per case | Units per case | Unit cost (PHP) 2015 | Cost per ave case (PHP) 2015 |
| Oxygen, 1000 L, primarily with oxygen cylinders | 100 | Changed price to minimum retail price of 315 based from costing survey, reduced days to 5 reflecting adequate antibiotic treatment  Accepted default of 2 liters per minute | 0.002 | 1,440 | 5 | 14.4 | 315.00 | 4,536.00 |
| Pulse oximeter | 100 | Pulse oximetry is useful for tracking and/or adjusting supplemental oxygen therapy (GOLD 2014)  Estimated to be 1,500 for 5,000 patients’ worth | 0.0002 | 1 | 5 | 5 | 1,500 | 0.03 |
| Arterial blood gas (ABG) | 100 | Once oxygen is started, arterial blood gases should be checked 30--60 minutes later to ensure satisfactory oxygenation  without carbon dioxide retention or acidosis  Requirement for discharge from hospital is that ABG is stable for the last 12-24 hours (GOLD 2014)  Based price from [Philippine Lung Center](http://lcp.gov.ph/images/Transparency/Citizens_Charter_revised2013.pdf) | 1 | 1 | 5 | 5 | 680 | 3,400 |
| ECG | 100 | May aid in the diagnosis of co-existing cardiac problems (GOLD 2014)  Based price from Philippine Lung Center | 1 | 1 | 1 | 1 | 365 | 365 |
| MEDICAL PERSONNEL | | | | | | | | |
| Staff type | % | Note | Minutes | | # of days/visits | | | Total |
| Generalist | 100 | See comment in Table 1.12 | 20 | | 2 | | | 40 |
| Nurse | 100 |  | 10 | | 5 | | | 50 |
| OUTPATIENT VISITS AND INPATIENT DAYS | | | | | | | | |
| Type | % | Note | Units per case | | | | |  |
| Outpatient visit | 100 | See comment in Table 1.12 | 5 | | | | | 5 |

### Delivery Channels

Table 1.15. Percent of interventions delivered at different levels

|  | Community | Outreach | Clinic | Hospital | Notes |
| --- | --- | --- | --- | --- | --- |
| COPD: Smoking cessation |  |  | 50 | 50 | PCCP CPG recommends that a doctor at the outpatient level delivers the three minute consult  Spirometry can also be done in both the clinic and hospital level |
| COPD: Inhaled salbutamol | 20 |  | 50 | 30 | Estimated delivery mix given that intervention is preferably delivered at lower levels of care.  Salbutamol assumed to be available at barangay health stations |
| COPD: Add-on Long-acting Bronchodilators for Moderate COPD |  |  | 70 | 30 |  |
| COPD: Ipratropium inhaler |  |  | 70 | 30 |  |
| COPD: Exacerbation treatment with antibiotics |  |  |  | 100 | PCCP CPG recommends that exacerbation treatment be delivered at inpatient level |
| COPD: Exacerbation treatment with oral prednisolone |  |  |  | 100 |  |
| COPD: Exacerbation treatment with oxygen |  |  |  | 100 |  |

#

# 2. Diabetes

## EPIDEMIOLOGY

### DISMOD II Inputs

Associated health states were modeled from DISMOD II using the following inputs:

Table 2.1. Diabetes (Uncomplicated) inputs for DISMOD II

| Age group | Prevalence (per 1,000) | | Relative risk of mortality | | Remission |
| --- | --- | --- | --- | --- | --- |
|  | From the [National Nutrition Survey 2008](https://www.slideshare.net/isiptan/philippine-clinical-practice-guidelines-for-the-diagnosis-and-management-of-type-2-diabetes-mellitus) | | From [ADVANCE Tria](http://care.diabetesjournals.org/content/32/suppl_2/S357.full)l, but denominator adjusted to mortality across population instead of unexposed | | Diabetic patients do not recover from diabetes |
|  | Male | Female | Male | Female | Male/Female |
| 0 to 24 | 9 | | 1 | 1 | 0 |
| 5 to 9 |  |  |  |  | 0 |
| 10 to 14 |  |  |  |  | 0 |
| 15 to 19 |  |  |  |  | 0 |
| 20 to 24 |  |  |  |  | 0 |
| 25 to 29 |  |  | 2.39 | 2.39 | 0 |
| 30 to 34 | 38 | | 2.37 | 2.37 | 0 |
| 35 to 39 |  |  | 2.35 | 2.34 | 0 |
| 40 to 44 | 82 | | 2.32 | 2.31 | 0 |
| 45 to 49 |  |  | 2.28 | 2.27 | 0 |
| 50 to 54 | 130 | | 2.24 | 2.23 | 0 |
| 55 to 59 |  |  | 2.21 | 2.19 | 0 |
| 60 to 64 | 159 | | 1.68 | 1.67 | 0 |
| 65 to 69 |  |  | 1.67 | 1.66 | 0 |
| 70 to 74 | 118 | | 1.67 | 1.66 | 0 |
| 75 to 79 |  |  | 1.35 | 1.35 | 0 |
| 80 above |  |  | 1.33 | 1.33 | 0 |

Table 2.2. Amputation from diabetes inputs for DISMOD II

| Age group | Prevalence (per 1,000) | | Relative risk of mortality | | Remission |
| --- | --- | --- | --- | --- | --- |
|  | Prevalent cases from 2010 GBD modeled for Philippines  Denominator from NNS 2008 and PSA population projection | | Adjusted from RR mortality of uncomplicated diabetes:  RR(sequelae) =  (RR(RR-1)*2)+1 | | No recovery from amputation |
|  | Male | Female | Male | Female | Male/Female |
| 0 to 4 | 0 | 0 | 1 | 1 | 0 |
| 5 to 9 | 0 | 0 |  |  | 0 |
| 10 to 14 | 0 | 0 |  |  | 0 |
| 15 to 19 | 1.5299 | 0.9770 |  |  | 0 |
| 20 to 24 | 15.5277 | 9.6802 |  |  | 0 |
| 25 to 29 | 50.5601 | 31.2602 | 7.6378 | 7.6375 | 0 |
| 30 to 34 | 26.8890 | 16.9184 | 7.4900 | 7.4818 | 0 |
| 35 to 39 | 40.3667 | 25.5194 | 7.3209 | 7.2988 | 0 |
| 40 to 44 | 21.9029 | 14.1255 | 7.1179 | 7.0721 | 0 |
| 45 to 49 | 22.0182 | 14.5011 | 6.8481 | 6.7780 | 0 |
| 50 to 54 | 10.7257 | 7.1247 | 6.5797 | 6.4690 | 0 |
| 55 to 59 | 12.1483 | 8.3514 | 6.3288 | 6.1968 | 0 |
| 60 to 64 | 14.4257 | 9.7377 | 3.2640 | 3.2294 | 0 |
| 65 to 69 | 10.6423 | 6.2427 | 3.2367 | 3.1991 | 0 |
| 70 to 74 |  |  | 3.2180 | 3.2056 | 0 |
| 75 to 79 |  |  | 1.9456 | 1.9476 | 0 |
| 80 above |  |  | 1.8742 | 1.8698 | 0 |

Table 2.3. Blindness from diabetes inputs for DISMOD II

| Age group | Prevalence (per 1,000) | | Relative risk of mortality | | Remission |
| --- | --- | --- | --- | --- | --- |
|  | Prevalent cases from 2010 GBD modeled for Philippines  Denominator from NNS 2008 and PSA population projection | | Adjusted from RR mortality of uncomplicated diabetes:  RR(sequelae) =  (RR(RR-1)*2)+1 | | No recovery from amputation |
|  | Male | Female | Male | Female | Male/Female |
| 0 to 4 | 0.0272 | 0.0759 | 1 | 1 | 0 |
| 5 to 9 | 2.4152 | 7.0149 |  |  | 0 |
| 10 to 14 | 14.9081 | 45.8802 |  |  | 0 |
| 15 to 19 | 19.4490 | 56.4459 |  |  | 0 |
| 20 to 24 | 13.9048 | 36.1651 |  |  | 0 |
| 25 to 29 | 17.7240 | 41.3243 | 7.6378 | 7.6375 | 0 |
| 30 to 34 | 5.6673 | 11.0314 | 7.4900 | 7.4818 | 0 |
| 35 to 39 | 7.1900 | 13.4696 | 7.3209 | 7.2988 | 0 |
| 40 to 44 | 4.5529 | 8.5404 | 7.1179 | 7.0721 | 0 |
| 45 to 49 | 6.1244 | 10.8201 | 6.8481 | 6.7780 | 0 |
| 50 to 54 | 5.1784 | 8.4524 | 6.5797 | 6.4690 | 0 |
| 55 to 59 | 7.6776 | 12.0131 | 6.3288 | 6.1968 | 0 |
| 60 to 64 | 7.8074 | 11.4032 | 3.2640 | 3.2294 | 0 |
| 65 to 69 | 4.5142 | 5.7162 | 3.2367 | 3.1991 | 0 |
| 70 to 74 |  |  | 3.2180 | 3.2056 | 0 |
| 75 to 79 |  |  | 1.9456 | 1.9476 | 0 |
| 80 above |  |  | 1.8742 | 1.8698 | 0 |

### Health State: Disease free/Susceptible to Transition: Incidence

Table 2.4. **Male** Diabetes Inputs for GCEA. Those in gray mean that defaults were accepted.

| Age group | Disability weight | Prevalence  (per 1,000) | Transitions - Incidence | |
| --- | --- | --- | --- | --- |
|  |  |  | Rates (per 1,000) | To health state |
| 0 to 4 | 0.03 | Grayed out (Prevalence rate changed in “Health State: Diabetes”) | 0.0061 | Diabetes |
| 5 to 9 | 0.05 |  | 0.0061 | Diabetes |
| 10 to 14 | 0.06 |  | 0.0061 | Diabetes |
| 15 to 19 | 0.07 |  | 0.0061 | Diabetes |
| 20 to 24 | 0.09 |  | 0.0061 | Diabetes |
| 25 to 29 | 0.10 |  | 1.274 | Diabetes |
| 30 to 39 | 0.12 |  | 4.0492 | Diabetes |
| 40 to 49 | 0.15 |  | 5.851 | Diabetes |
| 50 to 59 | 0.18 |  | 4.5668 | Diabetes |
| 60 to 69 | 0.21 |  | 5.5699 | Diabetes |
| 70 to 79 | 0.23 |  | 0.157 | Diabetes |
| 80 to 100 | 0.27 |  | 3.8347 | Diabetes |

Table 2.5. **Female** Diabetes Inputs for GCEA. Those in gray mean that defaults were accepted.

| Age group | Disability weight | Prevalence  (per 1,000) | Transitions - Incidence | |
| --- | --- | --- | --- | --- |
|  |  |  | Rates (per 1,000) | To health state |
| 0 to 4 | 0.03 | Grayed out (Prevalence rate changed in “Health State: Diabetes”) | 0.0061 | Diabetes |
| 5 to 9 | 0.04 |  | 0.0061 | Diabetes |
| 10 to 14 | 0.06 |  | 0.0061 | Diabetes |
| 15 to 19 | 0.07 |  | 0.0061 | Diabetes |
| 20 to 24 | 0.09 |  | 0.0061 | Diabetes |
| 25 to 29 | 0.10 |  | 1.270 | Diabetes |
| 30 to 39 | 0.12 |  | 4.0337 | Diabetes |
| 40 to 49 | 0.15 |  | 5.7502 | Diabetes |
| 50 to 59 | 0.18 |  | 4.1563 | Diabetes |
| 60 to 69 | 0.20 |  | 4.9666 | Diabetes |
| 70 to 79 | 0.23 |  | 0.1183 | Diabetes |
| 80 to 100 | 0.27 |  | 1.3611 | Diabetes |

### Health State: Diabetes to Transition: Incidence

Table 2.6. **Male** Diabetes Sequelae Inputs for GCEA. Those in gray mean that defaults were accepted.

| Age group | Disability weight | Prevalence  (per 1,000) | Transitions - Incidence | | Transitions - Incidence | |
| --- | --- | --- | --- | --- | --- | --- |
|  |  |  | Rates  (per 1,000) | To health state | Rates  (per 1,000) | To health state |
| 0 to 4 | 0.03 | 9.0148 | 0.2468 | Blindness | 0.0004 | Lower Ext. Amp. |
| 5 to 9 | 0.03 | 9.0446 | 2.1632 | Blindness | 0.0084 | Lower Ext. Amp. |
| 10 to 14 | 0.03 | 9.0748 | 6.1481 | Blindness | 0.1754 | Lower Ext. Amp. |
| 15 to 19 | 0.03 | 9.105 | 5.6953 | Blindness | 0.4407 | Lower Ext. Amp. |
| 20 to 24 | 0.03 | 9.1329 | 0.8993 | Blindness | 3.6716 | Lower Ext. Amp. |
| 25 to 29 | 0.03 | 11.0334 | 0.006 | Blindness | 7.8685 | Lower Ext. Amp. |
| 30 to 39 | 0.03 | 36.3173 | 0.0057 | Blindness | 1.6513 | Lower Ext. Amp. |
| 40 to 49 | 0.03 | 74.405 | 0.0056 | Blindness | 0.006 | Lower Ext. Amp. |
| 50 to 59 | 0.03 | 123.9864 | 0.0058 | Blindness | 0.0059 | Lower Ext. Amp. |
| 60 to 69 | 0.03 | 154.3135 | 0.0058 | Blindness | 0.0136 | Lower Ext. Amp. |
| 70 to 79 | 0.03 | 148.1784 | 0.0063 | Blindness | 0.3629 | Lower Ext. Amp. |
| 80 to 100 | 0.03 | 119.8472 | 0.2481 | Blindness | 1.3181 | Lower Ext. Amp. |

Table 2.7. **Female** Diabetes Sequelae Inputs for GCEA. Those in gray mean that defaults were accepted.

| Age group | Disability weight | Prevalence  (per 1,000) | Transitions - Incidence | | Transitions - Incidence | |
| --- | --- | --- | --- | --- | --- | --- |
|  |  |  | Rates  (per 1,000) | To health state | Rates  (per 1,000) | To health state |
| 0 to 4 | 0.03 | 9.0148 | 0.0856 | Blindness | 0.003 | Lower Ext. Amp. |
| 5 to 9 | 0.03 | 9.0446 | 0.7364 | Blindness | 0.0065 | Lower Ext. Amp. |
| 10 to 14 | 0.03 | 9.0748 | 2.0426 | Blindness | 0.1225 | Lower Ext. Amp. |
| 15 to 19 | 0.03 | 9.1051 | 1.8798 | Blindness | 0.2571 | Lower Ext. Amp. |
| 20 to 24 | 0.03 | 9.1341 | 0.2979 | Blindness | 2.2115 | Lower Ext. Amp. |
| 25 to 29 | 0.03 | 11.037 | 0.006 | Blindness | 4.8517 | Lower Ext. Amp. |
| 30 to 39 | 0.03 | 36.3873 | 0.0057 | Blindness | 1.0186 | Lower Ext. Amp. |
| 40 to 49 | 0.03 | 74.3782 | 0.0056 | Blindness | 0.0059 | Lower Ext. Amp. |
| 50 to 59 | 0.03 | 124.1685 | 0.0059 | Blindness | 0.0058 | Lower Ext. Amp. |
| 60 to 69 | 0.03 | 156.2927 | 0.0094 | Blindness | 0.0059 | Lower Ext. Amp. |
| 70 to 79 | 0.03 | 160.901 | 0.1596 | Blindness | 0.0066 | Lower Ext. Amp. |
| 80 to 100 | 0.03 | 134.4066 | 0.5583 | Blindness | 0.3121 | Lower Ext. Amp. |

### Health State: Diabetes to Transition: Case-fatality rate

Table 2.8. **Male** Diabetes Inputs for GCEA. Those in gray mean that defaults were accepted.

| Age group | Disability weight | Prevalence  (per 1,000) | Transitions - Case-fatality rate | |
| --- | --- | --- | --- | --- |
|  |  |  | Rates (per 1,000) | To health state |
| 0 to 4 | 0.03 | 9.0148 | 0.0061 | Deceased |
| 5 to 9 | 0.03 | 9.0446 | 0.0061 | Deceased |
| 10 to 14 | 0.03 | 9.0748 | 0.0061 | Deceased |
| 15 to 19 | 0.03 | 9.105 | 0.0084 | Deceased |
| 20 to 24 | 0.03 | 9.1329 | 0.1255 | Deceased |
| 25 to 29 | 0.03 | 11.0334 | 0.5467 | Deceased |
| 30 to 39 | 0.03 | 36.3173 | 1.505 | Deceased |
| 40 to 49 | 0.03 | 74.405 | 3.2652 | Deceased |
| 50 to 59 | 0.03 | 123.9864 | 7.6417 | Deceased |
| 60 to 69 | 0.03 | 154.3135 | 14.7212 | Deceased |
| 70 to 79 | 0.03 | 148.1784 | 28.4419 | Deceased |
| 80 to 100 | 0.03 | 119.8472 | 45.2387 | Deceased |

Table 2.9. **Female** Diabetes Inputs for GCEA. Those in gray mean that defaults were accepted.

| Age group | Disability weight | Prevalence  (per 1,000) | Transitions - Case-fatality rate | |
| --- | --- | --- | --- | --- |
|  |  |  | Rates (per 1,000) | To health state |
| 0 to 4 | 0.03 | 9.0148 | 0.0061 | Deceased |
| 5 to 9 | 0.03 | 9.0446 | 0.006 | Deceased |
| 10 to 14 | 0.03 | 9.0748 | 0.006 | Deceased |
| 15 to 19 | 0.03 | 9.1051 | 0.007 | Deceased |
| 20 to 24 | 0.03 | 9.1341 | 0.0522 | Deceased |
| 25 to 29 | 0.03 | 11.037 | 0.2629 | Deceased |
| 30 to 39 | 0.03 | 36.3873 | 0.9495 | Deceased |
| 40 to 49 | 0.03 | 74.3782 | 2.2052 | Deceased |
| 50 to 59 | 0.03 | 124.1685 | 4.5304 | Deceased |
| 60 to 69 | 0.03 | 156.2927 | 7.166 | Deceased |
| 70 to 79 | 0.03 | 160.901 | 14.5273 | Deceased |
| 80 to 100 | 0.03 | 134.4066 | 32.8788 | Deceased |

### Health State: Diabetic Blindness to Transition: Case-fatality rate

Table 2.10. **Male** Diabetic Blindness Inputs for GCEA. Those in gray mean that defaults were accepted.

| Age group | Disability weight | Prevalence  (per 1,000) | Transitions - Case-fatality rate | |
| --- | --- | --- | --- | --- |
|  |  |  | Rates (per 1,000) | To health state |
| 0 to 4 | 0.34 | 0.1171 | 0.0061 | Deceased |
| 5 to 9 | 0.34 | 1.8798 | 0.0061 | Deceased |
| 10 to 14 | 0.34 | 8.7549 | 0.0061 | Deceased |
| 15 to 19 | 0.34 | 19.7235 | 0.0082 | Deceased |
| 20 to 24 | 0.34 | 24.7663 | 0.1007 | Deceased |
| 25 to 29 | 0.34 | 25.0735 | 1.6015 | Deceased |
| 30 to 39 | 0.34 | 24.142 | 8.7415 | Deceased |
| 40 to 49 | 0.34 | 20.9718 | 22.4169 | Deceased |
| 50 to 59 | 0.34 | 15.1102 | 44.5631 | Deceased |
| 60 to 69 | 0.34 | 9.3283 | 56.4501 | Deceased |
| 70 to 79 | 0.34 | 5.2012 | 81.9305 | Deceased |
| 80 to 100 | 0.34 | 4.4495 | 120.5021 | Deceased |

Table 2.11. **Female** Diabetic Blindness Inputs for GCEA. Those in gray mean that defaults were accepted.

| Age group | Disability weight | Prevalence  (per 1,000) | Transitions - Case-fatality rate | |
| --- | --- | --- | --- | --- |
|  |  |  | Rates (per 1,000) | To health state |
| 0 to 4 | 0.34 | 0.33 | 0.0061 | Deceased |
| 5 to 9 | 0.34 | 5.4591 | 0.006 | Deceased |
| 10 to 14 | 0.34 | 25.7152 | 0.006 | Deceased |
| 15 to 19 | 0.34 | 58.0543 | 0.0068 | Deceased |
| 20 to 24 | 0.34 | 72.6409 | 0.0375 | Deceased |
| 25 to 29 | 0.34 | 73.5767 | 0.9663 | Deceased |
| 30 to 39 | 0.34 | 71.6598 | 6.7049 | Deceased |
| 40 to 49 | 0.34 | 63.3894 | 22.0294 | Deceased |
| 50 to 59 | 0.34 | 46.2036 | 41.8734 | Deceased |
| 60 to 69 | 0.34 | 29.7958 | 54.7429 | Deceased |
| 70 to 79 | 0.34 | 15.1821 | 80.1765 | Deceased |
| 80 to 100 | 0.34 | 7.3361 | 91.1755 | Deceased |

### Health State: Diabetic Lower Extremity Amputation to Transition: Case-fatality rate

Table 2.12. **Male** Diabetic Lower Extremity Amputation Inputs for GCEA. Those in gray mean that defaults were accepted.

| Age group | Disability weight | Prevalence  (per 1,000) | Transitions - Case-fatality rate | |
| --- | --- | --- | --- | --- |
|  |  |  | Rates (per 1,000) | To health state |
| 0 to 4 | 0.34 | 0.001 | 0.0001 | Deceased |
| 5 to 9 | 0.34 | 0.0077 | 0.001 | Deceased |
| 10 to 14 | 0.34 | 0.3898 | 0.0005 | Deceased |
| 15 to 19 | 0.34 | 1.7792 | 0.025 | Deceased |
| 20 to 24 | 0.34 | 9.7538 | 0.13 | Deceased |
| 25 to 29 | 0.34 | 40.0856 | 1.6034 | Deceased |
| 30 to 39 | 0.34 | 68.6318 | 7.1085 | Deceased |
| 40 to 49 | 0.34 | 63.5329 | 22.2709 | Deceased |
| 50 to 59 | 0.34 | 42.4107 | 62.9993 | Deceased |
| 60 to 69 | 0.34 | 22.7057 | 63.3868 | Deceased |
| 70 to 79 | 0.34 | 12.377 | 82.1547 | Deceased |
| 80 to 100 | 0.34 | 10.513 | 120.4836 | Deceased |

Table 2.13. **Female** Diabetic Lower Extremity Amputation Inputs for GCEA. Those in gray mean that defaults were accepted.

| Age group | Disability weight | Prevalence  (per 1,000) | Transitions - Case-fatality rate | |
| --- | --- | --- | --- | --- |
|  |  |  | Rates (per 1,000) | To health state |
| 0 to 4 | 0.34 | 0.0007 | 0.0001 | Deceased |
| 5 to 9 | 0.34 | 0.0059 | 0.009 | Deceased |
| 10 to 14 | 0.34 | 0.2787 | 0.0014 | Deceased |
| 15 to 19 | 0.34 | 1.1671 | 0.0156 | Deceased |
| 20 to 24 | 0.34 | 5.891 | 0.0494 | Deceased |
| 25 to 29 | 0.34 | 24.7046 | 0.8589 | Deceased |
| 30 to 39 | 0.34 | 43.261 | 4.3396 | Deceased |
| 40 to 49 | 0.34 | 41.7559 | 13.8639 | Deceased |
| 50 to 59 | 0.34 | 32.6504 | 36.5197 | Deceased |
| 60 to 69 | 0.34 | 22.438 | 41.3174 | Deceased |
| 70 to 79 | 0.34 | 13.6246 | 61.9457 | Deceased |
| 80 to 100 | 0.34 | 7.5374 | 87.4983 | Deceased |

## INTERVENTION COSTING

### Target Population

All interventions address people with diabetes - defaults retained

### Population-in-Need

Across all years:

Table 2.14. Population-in-need inputs in GCEA.

| Intervention | Default | Changed | Rationale / Sources |
| --- | --- | --- | --- |
| Standard glycemic control | 100% | 100% | Standard interventions for all diabetic patients |
| Intensive glycemic control | 100% | 100% |  |
| Retinopathy screening and photocoagulation | 100% | 100% |  |
| Neuropathy screening and preventive foot care | 100% | 100% |  |

###

### Intervention Coverage

This pertains to the “Baseline Coverage from impact module (2016)” column:

Table 2.15. Intervention coverage inputs in GCEA.

| Intervention | Default | Changed | Rationale / Sources |
| --- | --- | --- | --- |
| Standard glycemic control | 20% | 10.3% | From expert survey: 57.14%, multiplied by 20% as this is the expected true prevalence rate of diabetes in the Philippines according to UNITE for Diabetes Philippines |
| Intensive glycemic control | 5% | 7.3% | From expert survey: 36.43%, multiplied by 20% as this is the expected true prevalence rate of diabetes in the Philippines according to UNITE for Diabetes Philippines |
| Retinopathy screening and photocoagulation | 10% | 8.5% | From expert survey: 49.29%, multiplied by 20% as this is the expected true prevalence rate of diabetes in the Philippines according to UNITE for Diabetes Philippines |
| Neuropathy screening and preventive foot care | 10% | 6.8% | From expert survey: 34.05%, multiplied by 20% as this is the expected true prevalence rate of diabetes in the Philippines according to UNITE for Diabetes Philippines |

###

### Treatment Inputs

Table 2.16. Treatment inputs for **Standard glycemic control** in GCEA

| **Intervention Name: Standard glycemic control** | | | | | | | | |
| --- | --- | --- | --- | --- | --- | --- | --- | --- |
| DRUGS AND SUPPLIES | | | | | | | | |
| Drug/supply | % rec asp of treat | Note | # units | Times per day | Days per case | Units per case | Unit cost (PHP) 2015 | Cost per ave case (PHP) 2015 |
| Insulin |  | Removed, UNITE guideline suggest patients start on oral anti-diabetic (OAD) first before considering insulin (when glucose is inadequately controlled) |  |  |  |  |  |  |
| Home glucose monitoring |  | Removed; see capillary blood glucose test |  |  |  |  |  |  |
| Glibenclamide, 5 mg tab | 40.0 | As per UNITE guidelines: additional drug for combination therapy if metformin is not enough (most effective in lowering HbA1c among antidiabetic alternatives aside from metformin) - combination therapy required for those HbA1c > 9%  Changed % to 40 based on an epidemiological study done by Matsuoka (2002) in the Philippines  Changed dosage to 2.5 mg once or [twice a day](https://www.nps.org.au/__data/assets/pdf_file/0005/14693/metformin_glibenclamide.pdf). Since DPRI only has available price for 5 mg, number of unit became 0.5  Changed price based on DPRI (0.27) + 30% markup (0.351) | 0.5 | 1.5 | 365 | 273.75 | 0.351 | 96.09 |
| Metformin, 500 mg tab | 59.0 | As per UNITE guidelines: primary drug of choice for patients with high HBA1c or FBS (8-9%)  Changed % to 59 based on an epidemiological study done by [Lantion-Ang (2000)](https://www.ncbi.nlm.nih.gov/pubmed/11024581) in the Philippines  Changed dosage to 500 mg, once daily  Changed price based on DRPI (0.78) + 30% markup (1.01) | 1.0 | 1 | 365 | 365 | 1.01 | 368.65 |
| Monthly monitoring (removed due to duplicity above) | | | | | | | | |
| Home glucose monitoring | 100.0 | Removed due to duplicity above |  |  |  |  |  |  |
| Testing | | | | | | | | |
| HbA1c test | 100.0 | Input for hospital level only (assumption that HbA1c is not available at lower levels of care)  UNITE guidelines recommend checking HbA1c levels every 3 to 6 months  Changed price based on minimum price from costing survey | 1.0 | 1 | 3 | 3 | 700 | 2,100.00 |
| Blood glucose level test | 100.0 | Input for clinic and hospital levels only (assumption that both not available at community level)  Dr. Isip Tan’s [presentation](https://www.slideshare.net/isiptan/philippine-clinical-practice-guidelines-for-the-diagnosis-and-management-of-type-2-diabetes-mellitus) (aligned with UNITE guidelines) recommend FBS every 2 to 4 weeks  Changed price based on minimum price from costing survey | 1.0 | 1 | 17 | 17 | 140 | 2,380.00 |
| Capillary test | 100.0 | Dr Isip Tan’s presentation recommend (aligned with UNITE guidelines) 2x a week  According to Philippine Medical Supplies, a glucometer costs 1,500. Assuming it takes 5 min/test on an 8 hour workday for 3 years, each case will cost 0.014  One lancet assumed to be 0.8 (160 per 200 pc). Each strip costing 30 pesos. Batteries included in glucometer costing. | 1.0 | 1 | 104 | 104 | 30.814 | 3,204.24 |
| Blood test: Test for fasting lipid profile | 100.0 | Removed, not an essential intervention under glycemic control as per UNITE guidelines |  |  |  |  |  |  |
| Urine test: Test for urine albumin excretion and albumin to creatinine ratio | 100.0 | Removed, not an essential intervention under glycemic control as per UNITE guidelines |  |  |  |  |  |  |
| MEDICAL PERSONNEL | | | | | | | | |
| Staff type | % | Note | Minutes | | # of days/visits | | | Total |
| Nurses | 100.0 | Changed frequency of visit to reflect number of times nurse needs to assist patient in HbA1c/FBS | 10 | | 3 | | | 30 |
| Generalists | 100.0 | Changed frequency of visit to reflect number of times doctor needs to interpret FBS results  Input only for clinic level | 10 | | 3 | | | 30 |
| Other specialist doctors | 10.0 | Endocrinologist or internal medicine specialist; changed frequency of visit to reflect number of times doctor needs to interpret HbA1c results  Input only for hospital level | 10 | | 3 | | | 30 |
| OUTPATIENT VISITS AND INPATIENT DAYS | | | | | | | | |
| Type | % | Note | Units per case | | | | | Total |
| Outpatient visit | 100 | Changed frequency of visit to reflect number of times needed to do tests | 3 | | | | | 3 |

Table 2.17. Treatment inputs for **Intensive glycemic control** in GCEA

| **Intervention Name: Intensive glycemic control** | | | | | | | | |
| --- | --- | --- | --- | --- | --- | --- | --- | --- |
| DRUGS AND SUPPLIES | | | | | | | | |
| Drug/supply | % rec asp of treat | Note | # units | Times per day | Days per case | Units per case | Unit cost (PHP) 2015 | Cost per ave case (PHP) 2015 |
| Basal Insulin  (NPH/Isophane) | 47.0 | % default accepted because UNITE guidelines do not have intensive glycemic control guidelines - % came from ADVANCE trial of which Philippines is included  Basal insulin usually with metformin +/- other non-insulin agent  People with type  2 diabetes without history of hypoglycemia  or severe hypoglycemia may use  NPH safely at much lower cost  Isophane DPRI price+30% | 10 | 1 | 365 | 3650 | .21 | 766.50 |
| Home glucose monitoring |  | Removed; see capillary blood glucose test |  |  |  |  |  |  |
| Glibenclamide, 5 mg tab | 85.0 | % default accepted because UNITE guidelines do not have intensive glycemic control guidelines - % came from ADVANCE trial of which Philippines is included  Dosage retained as this was the mean dosage level among patients in the ADVANCE trial.  Changed price (see comment in Table 2.16) | 4.0 | 1 | 365 | 1,460 | 0.351 | 512.46 |
| Metformin, 500 mg tab | 67.0 | % default accepted because UNITE guidelines do not have intensive glycemic control guidelines - % came from ADVANCE trial of which Philippines is included  Dosage retained as this was the mean dosage level among patients in the ADVANCE trial.  Changed price and tablet form (see comment in Table 2.16), but retained overall dosage | 1.7 | 3 | 365 | 1,861.5 | 1.01 | 1,880.12 |
| Monthly monitoring (removed due to duplicity above)hly monitoring | | | | | | | | |
| Home glucose monitoring | 100.0 | Removed due to duplicity above |  |  |  |  |  |  |
| Testing | | | | | | | | |
| HbA1c test | 100.0 | See Table 2.16 for comment | 1.0 | 1 | 3 | 3 | 700 | 2,100.00 |
| Blood glucose level test | 100.0 | See Table 2.16 for comment | 1.0 | 1 | 17 | 17 | 140 | 2,380.00 |
| Capillary test | 100.0 | Patients on once daily insulin plus oral meds with high HbA1c are recommended to test at least twice daily including pre and postprandial. | 1.0 | 8 | 365 | 2920 | 30.814 | 89,976.88 |
| Blood test: Test for fasting lipid profile |  | Removed, see Table 2.16 for comment |  |  |  |  |  |  |
| Urine test: Test for urine albumin excretion and albumin to creatinine ratio |  | Removed, see Table 2.16 for comment |  |  |  |  |  |  |
| MEDICAL PERSONNEL | | | | | | | | |
| Staff type | % | Note | Minutes | | # of days/visits | | | Total |
| Nurses | 100.0 | See Table 2.16 for comment | 10 | | 3 | | | 30 |
| Generalists | 100.0 | See Table 2.16 for comment | 10 | | 3 | | | 30 |
| Other specialist doctors | 10.0 | See Table 2.16 for comment | 10 | | 3 | | | 30 |
| OUTPATIENT VISITS AND INPATIENT DAYS | | | | | | | | |
| Type | % | Note | Units per case | | | | | Total |
| Outpatient visit | 100 | See Table 2.16 for comment | 3 | | | | | 3 |

Table 2.18. Treatment inputs for **Retinopathy screening and photocoagulation** in GCEA

| **Intervention Name: Retinopathy screening and photocoagulation** | | | | | | | | |
| --- | --- | --- | --- | --- | --- | --- | --- | --- |
| DRUGS AND SUPPLIES | | | | | | | | |
| Drug/supply | % rec asp of treat | Note | # units | Times per day | Days per case | Units per case | Unit cost (PHP) 2015 | Cost per ave case (PHP) 2015 |
| Fluorescein drops | 100.0 | Default price kept, no other cost source available  According to WHO PEN guidelines, screening for retinopathy is done every 2 years. | 2.0 | 1 | 0.5 | 2 | 4.02 | 8.04 |
| Portable slit lamp | 100.0 | Default price kept, no other cost source available  Cost $4,000; one lamp per 5,000  See comment in previous box | 1.0 | 1 | 0.5 | 1 | 32.83 | 32.83 |
| Laser photocoagulation | 12.0 | Changed % to 12, as this is the expected prevalence of retinopathy according to UNITE guidelines  Default price kept, no other cost source available | 1.0 | 1 | 2 | 2 | 0.00 | 0.00 |
| Tetracaine drops | 12% | Default price kept, no other cost source available | 1.0 | 1 | 2 | 2 | 0.55 | 0.06 |
| Antireflective lens | 5.0 | Default price kept, no other cost source available | 1.0 | 1 | 2 | 2 | 0.00 | 0.00 |
| MEDICAL PERSONNEL | | | | | | | | |
| Staff type | % | Note | Minutes | | # of days/visits | | | Total |
| Other specialist doctors | 100.0 | Ophthalmologist | 10 | | 1 | | | 10 |
| Follow-up | | | | | | | | |
| Other specialist doctors | 5.0 | Ophthalmologist | 20 | | 2 | | | 2 |
| OUTPATIENT VISITS AND INPATIENT DAYS | | | | | | | | |
| Type | % | Note | Units per case | | | | | Total |
| Screening | | | | | | | | |
| Outpatient visit | 100 | Default | 1 | | | | | 1 |
| Follow up | | | | | | | | |
| Outpatient visit | 5.0 | Default | 2 | | | | | 0.1 |

Table 2.19. Treatment inputs for **Neuropathy screening and preventive foot care** in GCEA

| **Intervention Name: Neuropathy screening and preventive foot care** | | | | | | | | |
| --- | --- | --- | --- | --- | --- | --- | --- | --- |
| DRUGS AND SUPPLIES | | | | | | | | |
| Drug/supply | % rec asp of treat | Note | # units | Times per day | Days per case | Units per case | Unit cost (PHP) 2015 | Cost per ave case (PHP) 2015 |
| Gloves, exam, latex, disposable | 100.0 | Changed price based on minimum cost from costing survey | 1.0 | 1 | 1 | 1 | 2.05 | 2.05 |
| Monofilament | 100.0 | Default price kept, no other cost source available | 1.0 | 1 | 1 | 1 | 39.50 | 39.50 |
| Protective shoes | 20.0 | Unit cost defaults kept because protective shoes and insoles differ in quality and standard  % changed to 20 based from a study by [Fojas (2009)](https://www.pcp.org.ph/documents/PJIM/Volume%2047%20(2009)/Number%203%20(May-June)/2-Complications%20and%20Cardiovascular.pdf) in the Philippines | 1.0 | 1 | 1 | 1 | 5,530.57 | 5,530.57 |
| Insoles | 20.0 |  | 1.0 | 1 | 1 | 1 | 987.60 | 987.60 |
| MEDICAL PERSONNEL | | | | | | | | |
| Staff type | % | Note | Minutes | | # of days/visits | | | Total |
| Other specialist doctors | 100.0 | Neurologist | 20 | | 1 | | | 20 |
| Follow-up | | | | | | | | |
| Other specialist doctors | 10.0 | Neurologist | 20 | | 3 | | | 6 |
| OUTPATIENT VISITS AND INPATIENT DAYS | | | | | | | | |
| Type | % | Note | Units per case | | | | | Total |
| Screening | | | | | | | | |
| Outpatient visit | 100 | Default | 1 | | | | | 1 |
| Follow up | | | | | | | | |
| Outpatient visit | 10 | Default | 3 | | | | | 0.3 |

### Delivery Channels

Table 2.20. Percent of interventions delivered at different levels

|  | Community | Outreach | Clinic | Hospital | Notes |
| --- | --- | --- | --- | --- | --- |
| Standard glycemic control | 20 |  | 50 | 30 | Rough estimates only |
| Intensive glycemic control | 20 |  | 50 | 30 |  |
| Retinopathy screening and photocoagulation |  |  | 0 | 100 | Assumption that specialists cannot be found at MHO level |
| Neuropathy screening and preventive foot care |  |  | 0 | 100 |  |

# 3. CVD/Diabetes Screening, CVD Management

CVD Screening not included in the model as no impact module is linked yet but defaults were changed regardless.

## EPIDEMIOLOGY

Because of the lack of data on screening prevalence, defaults accepted in the tool.

## INTERVENTION COSTING

### Target Population

Defaults retained

### Population-in-Need

Defaults retained - Southeast Asian estimates

### Intervention Coverage

This pertains to the “Baseline Coverage from impact module (2016)” column:

Table 3.1. Intervention coverage inputs in GCEA

| Intervention | Default | Changed | Rationale / Sources |
| --- | --- | --- | --- |
| Screening for risk of CVD/diabetes | 0 | 41.14% | Expert survey result (weighted according to certainty of the expert’s estimate) |
| Follow-up care for those at low risk of CVD/diabetes (absolute risk: 10-20%) | 0 | 41.33% |  |
| Treatment for those with very high cholesterol but low absolute risk of CVD/diabetes (<20%) | 0 | 55.07% |  |
| Treatment for those with high blood pressure but low absolute risk of CVD/diabetes (<20%) | 0 | 61.51% |  |
| Treatment for those with absolute risk of CVD/diabetes (20-30%) | 0 | 51.50% |  |
| Treatment for those with high absolute risk of CVD/diabetes (>30%) | 0 | 62.83% |  |
| Treatment of new cases of acute myocardial infarction (AMI) with aspirin | 5 | 68.75% |  |
| Treatment of cases with established ischaemic heart disease (IHC) and post MI | 5 | 57.49% |  |
| Treatment for those with established cerebrovascular disease and post stroke | 5 | 55.91% |  |
| Treatment of cases with rheumatic heart disease (with benzathine penicillin) | 0 | 52.50% |  |

### Treatment Inputs

Table 3.2. Treatment inputs for **Screening for risk of CVD/diabetes** in GCEA

| **Intervention Name: Screening for risk of CVD/diabetes** | | | | | | | | |
| --- | --- | --- | --- | --- | --- | --- | --- | --- |
| DRUGS AND SUPPLIES | | | | | | | | |
| Drug/supply | % rec asp of treat | Note | # units | Times per day | Days per case | Units per case | Unit cost (PHP) 2015 | Cost per ave case (PHP) 2015 |
| Fasting blood glucose test | 100 | Based on Philippine PEN guidelines: Changed test to more specific FBS, changed % to 100  Changed price based on lowest price in costing survey | 1.0 | 1 | 1 | 1 | 20 | 20 |
| Cholesterol test | 100 | Based on Philippine PEN guidelines: Changed % to 100  Changed price based on lowest price in costing survey | 1.0 | 1 | 1 | 1 | 100 | 100 |
| Urine analysis | 100 | Based on Philippine PEN guidelines: Changed % to 100  Changed price based on lowest price in costing survey | 1.0 | 1 | 1 | 1 | 75 | 75 |
| Urine sugar analysis |  | Removed, assumed included in urine analysis |  |  |  |  |  |  |
| MEDICAL PERSONNEL | | | | | | | | |
| Staff type | % | Note | Minutes | | # of days/visits | | | Total |
| Nurse | 100 | Changed time to 10 minutes | 10 | | 1 | | | 10 |
| Generalist | 100 | To interpret results | 10 | | 1 | | | 10 |
| OUTPATIENT VISITS AND INPATIENT DAYS | | | | | | | | |
| Type | % | Note | Units per case | | | | | Total |
| Outpatient visit | 100 | Default | 1 | | | | | 1 |

Table 3.3. Treatment inputs for **Follow-up care for those at low risk of CVD/diabetes (absolute risk: 10-20%)** in GCEA

| **Intervention Name: Follow-up care for those at low risk of CVD/diabetes (absolute risk: 10-20%)** | | | | | | | | |
| --- | --- | --- | --- | --- | --- | --- | --- | --- |
| DRUGS AND SUPPLIES | | | | | | | | |
| Drug/supply | % rec asp of treat | Note | # units | Times per day | Days per case | Units per case | Unit cost (PHP) 2015 | Cost per ave case (PHP) 2015 |
| Fasting blood glucose test | 100 | See comment in Table 3.2  According to Philippine PEN guidelines, follow up every three month until targets are met | 1.0 | 1 | 4 | 1 | 20 | 80 |
| Cholesterol test | 100 |  | 1.0 | 1 | 4 | 1 | 100 | 400 |
| Urine analysis | 100 |  | 1.0 | 1 | 4 | 1 | 75 | 300 |
| Urine sugar analysis |  | Removed, assumed included in urine analysis |  |  |  |  |  |  |
| MEDICAL PERSONNEL | | | | | | | | |
| Staff type | % | Note | Minutes | | # of days/visits | | | Total |
| Nurse | 100 | See comment in Table 3.2 | 10 | | 4 | | | 40 |
| Generalist | 100 |  | 10 | | 4 | | | 40 |
| OUTPATIENT VISITS AND INPATIENT DAYS | | | | | | | | |
| Type | % | Note | Units per case | | | | | Total |
| Outpatient visit | 100 |  | 4 | | | | | 4 |

Table 3.4. Treatment inputs for **Treatment for those with very high cholesterol but low absolute risk of CVD/diabetes (<20%)** in GCEA

| **Intervention Name: Treatment for those with very high cholesterol but low absolute risk of CVD/diabetes (<20%)** | | | | | | | | |
| --- | --- | --- | --- | --- | --- | --- | --- | --- |
| DRUGS AND SUPPLIES | | | | | | | | |
| Drug/supply | % rec asp of treat | Note | # units | Times per day | Days per case | Units per case | Unit cost (PHP) 2015 | Cost per ave case (PHP) 2015 |
| Simvastatin, 10 mg | 100 | Changed dosage to 10 mg to reflect 2015 PHA guidelines (minimum dose)  Only simvastatin was included in the DPRI, also known to be more frequently prescribed than other statins  Changed price according to DPRI (1.85) + 30% markup (2.405) | 1 | 1 | 365 | 365 | 2.405 | 877.83 |
| Fasting blood glucose test | 100 | See comment in Table 3.2  According to Philippine PEN guidelines, follow up every three month until targets are met | 1.0 | 1 | 4 | 1 | 20 | 80 |
| Cholesterol test | 100 |  | 1.0 | 1 | 4 | 1 | 100 | 400 |
| Urine analysis | 100 |  | 1.0 | 1 | 4 | 1 | 75 | 300 |
| Urine sugar analysis |  | Removed, assumed included in urine analysis |  |  |  |  |  |  |
| MEDICAL PERSONNEL | | | | | | | | |
| Staff type | % | Note | Minutes | | # of days/visits | | | Total |
| Generalist | 100 | See comment in Table 3.2 | 10 | | 4 | | | 40 |
| Nurse | 100 |  | 10 | | 4 | | | 40 |
| OUTPATIENT VISITS AND INPATIENT DAYS | | | | | | | | |
| Type | % | Note | Units per case | | | | | Total |
| Outpatient visit | 100 |  | 4 | | | | | 4 |

Table 3.5. Treatment inputs for **Treatment for those with high blood pressure but low absolute risk of CVD/diabetes (<20%)** in GCEA

| **Intervention Name: Treatment for those with high blood pressure but low absolute risk of CVD/diabetes (<20%)** | | | | | | | | |
| --- | --- | --- | --- | --- | --- | --- | --- | --- |
| DRUGS AND SUPPLIES | | | | | | | | |
| Drug/supply | % rec asp of treat | Note | # units | Times per day | Days per case | Units per case | Unit cost (PHP) 2015 | Cost per ave case (PHP) 2015 |
| Drugs and supplies required per client | | | | | | | | |
| Hydrochlorothiazide, tablet 25 mg | [95.0](http://www.bmj.com/content/344/bmj.e607) | Changed price according to DPRI (3.10) + 30% markup (4.03) | 1.0 | 1 | 365 | 365 | 4.03 | 1,397.4025 |
| Enalapril, tablet, 5 mg | 40.0 | Changed dosage to the lowest recommended dose according to Philippine PEN guidelines  Changed price according to DPRI (3.85) + 30% markup (5.005) | 1.0 | 1 | 365 | 365 | 5.005 | 730.73 |
| Atenolol, tablets, 50 mg | 25.0 | Changed dosage to the lowest recommended dose according to Philippine PEN guidelines  Changed price according to DPRI (1.26) + 30% markup (21.638) | 1 | 1 | 365 | 365 | 1.638 | 328.8285 |
| Amlodipine, tablet, 5 mg | 25.0 | Changed dosage to the lowest recommended dose according to Philippine PEN guidelines  Changed price according to DPRI (0.61) + 30% markup (0.793) | 1 | 1 | 365 | 365 | 0.793 | 72.36125 |
| Laboratory tests per client | | | | | | | | |
| Fasting blood glucose test | 100 | See comment in Table 3.2  According to Philippine PEN guidelines, follow up every three month until targets are met | 1.0 | 1 | 4 | 1 | 20 | 80 |
| Cholesterol test | 100 |  | 1.0 | 1 | 4 | 1 | 100 | 400 |
| Urine analysis | 100 |  | 1.0 | 1 | 4 | 1 | 75 | 300 |
| Urine sugar analysis |  | Removed, assumed included in urine analysis |  |  |  |  |  |  |
| MEDICAL PERSONNEL | | | | | | | | |
| Staff type | % | Note | Minutes | | # of days/visits | | | Total |
| Generalist | 100 | See comment in Table 3.2 | 10 | | 4 | | | 40 |
| Nurse | 100 |  | 10 | | 4 | | | 40 |
| OUTPATIENT VISITS AND INPATIENT DAYS | | | | | | | | |
| Type | % | Note | Units per case | | | | | Total |
| Outpatient visit | 100 |  | 4 | | | | | 4 |

Table 3.6. Treatment inputs for **Treatment for those with absolute risk of CVD/diabetes (20-30%)** in GCEA

| **Intervention Name: Treatment for those with absolute risk of CVD/diabetes (20-30%)** | | | | | | | | |
| --- | --- | --- | --- | --- | --- | --- | --- | --- |
| DRUGS AND SUPPLIES | | | | | | | | |
| Drug/supply | % rec asp of treat | Note | # units | Times per day | Days per case | Units per case | Unit cost (PHP) 2015 | Cost per ave case (PHP) 2015 |
| Drugs and supplies required per client | | | | | | | | |
| Hydrochlorothiazide, tablet 25 mg | 95.0 | See comment in Table 3.4 | 1.0 | 1 | 365 | 365 | 4.03 | 1,397.4025 |
| Enalapril, tablet, 5 mg | 40.0 | See comment in Table 3.4 | 1.0 | 1 | 365 | 365 | 5.005 | 730.73 |
| Atenolol, tablets, 50 mg | 25.0 | See comment in Table 3.4 | 1 | 1 | 365 | 365 | 1.638 | 328.8285 |
| Amlodipine, tablet, 5 mg | 25.0 | See comment in Table 3.4 | 1 | 1 | 365 | 365 | 0.793 | 72.36125 |
| Simvastatin, 15 mg | 100 | See comment in Table 3.3 | 1 | 1 | 365 | 365 | 2.405 | 877.83 |
| Prednisolone, tablet, 5 mg |  | Removed because COPD oral medication not explicitly indicated in Philippine PEN guidelines |  |  |  |  |  |  |
| Laboratory tests per client | | | | | | | | |
| Fasting blood glucose test | 100 | See comment in Table 3.2  According to Philippine PEN guidelines, follow up every three month until targets are met | 1.0 | 1 | 4 | 1 | 20 | 80 |
| Cholesterol test | 100 |  | 1.0 | 1 | 4 | 1 | 100 | 400 |
| Urine analysis | 100 |  | 1.0 | 1 | 4 | 1 | 75 | 300 |
| Urine sugar analysis |  | Removed, assumed included in urine analysis |  |  |  |  |  |  |
| MEDICAL PERSONNEL | | | | | | | | |
| Staff type | % | Note | Minutes | | # of days/visits | | | Total |
| Generalist | 100 | Changed to 4 to reflect number of times lab tests have to be done | 10 | | 4 | | | 40 |
| Nurse | 7.0 | Management of complications due to diabetes | 20 | | 4 | | | 5.60 |
| OUTPATIENT VISITS AND INPATIENT DAYS | | | | | | | | |
| Type | % | Note | Units per case | | | | | Total |
| Treatment for all | | | | | | | | |
| Outpatient visit | 93 | Changed units to reflect changes in staffing and tests | 4 | | | | | 3.7 |
| Treatment of complications for those with diabetes | | | | | | | | |
| Outpatient visits | 7.0 | Changed units to reflect changes in staffing and tests | 8 | | | | | 0.56 |

Table 3.7. Treatment inputs for **Treatment for those with high absolute risk of CVD/diabetes (>30%)** in GCEA

| **Intervention Name: Treatment for those with high absolute risk of CVD/diabetes (>30%)** | | | | | | | | |
| --- | --- | --- | --- | --- | --- | --- | --- | --- |
| DRUGS AND SUPPLIES | | | | | | | | |
| Drug/supply | % rec asp of treat | Note | # units | Times per day | Days per case | Units per case | Unit cost (PHP) 2015 | Cost per ave case (PHP) 2015 |
| Drugs and supplies required per client | | | | | | | | |
| Hydrochlorothiazide, tablet 25 mg | 95.0 | See comment in Table 3.4 | 1.0 | 1 | 365 | 365 | 4.03 | 1,397.4025 |
| Enalapril, tablet, 5 mg | 40.0 | See comment in Table 3.4 | 1.0 | 1 | 365 | 365 | 5.005 | 730.73 |
| Atenolol, tablets, 50 mg | 25.0 | See comment in Table 3.4 | 1 | 1 | 365 | 365 | 1.638 | 328.8285 |
| Amlodipine, tablet, 5 mg | 25.0 | See comment in Table 3.4 | 1 | 1 | 365 | 365 | 0.793 | 72.36125 |
| Simvastatin, 15 mg | 100 | See comment in Table 3.3 | 1 | 1 | 365 | 365 | 2.405 | 877.83 |
| Prednisolone, tablet, 5 mg |  | Removed because COPD oral medication not explicitly indicated in Philippine PEN guidelines |  |  |  |  |  |  |
| Laboratory tests per client | | | | | | | | |
| Fasting blood glucose test | 100 | See comment in Table 3.2  According to Philippine PEN guidelines, follow up every three month until targets are met | 1.0 | 1 | 4 | 1 | 20 | 80 |
| Cholesterol test | 100 |  | 1.0 | 1 | 4 | 1 | 100 | 400 |
| Urine analysis | 100 |  | 1.0 | 1 | 4 | 1 | 75 | 300 |
| Urine sugar analysis |  | Removed, assumed included in urine analysis |  |  |  |  |  |  |
| MEDICAL PERSONNEL | | | | | | | | |
| Staff type | % | Note | Minutes | | # of days/visits | | | Total |
| Generalist | 100 | Changed to 4 to reflect number of times lab tests have to be done | 10 | | 4 | | | 40 |
| Nurse | 7.0 | Management of complications due to diabetes | 20 | | 4 | | | 5.60 |
| OUTPATIENT VISITS AND INPATIENT DAYS | | | | | | | | |
| Type | % | Note | Units per case | | | | | Total |
| Treatment for all | | | | | | | | |
| Outpatient visit | 93 | Changed units to reflect changes in staffing and tests | 4 | | | | | 3.7 |
| Treatment of complications for those with diabetes | | | | | | | | |
| Outpatient visits | 7.0 | Changed units to reflect changes in staffing and tests | 8 | | | | | 0.56 |

Table 3.8. Treatment inputs for **Treatment of new cases of acute myocardial infarction (AMI) with aspirin** in GCEA

| **Intervention Name: Treatment of new cases of acute myocardial infarction (AMI) with aspirin** | | | | | | | | |
| --- | --- | --- | --- | --- | --- | --- | --- | --- |
| DRUGS AND SUPPLIES | | | | | | | | |
| Drug/supply | % rec asp of treat | Note | # units | Times per day | Days per case | Units per case | Unit cost (PHP) 2015 | Cost per ave case (PHP) 2015 |
| Acetyl salysilic acid (aspirin), tablet, 80 6mg | 100 | Changed dosage to 80 mg as it is the lowest recommended daily dose for MI patients according to [PHA CAD](http://www.philheart.org/images/guidelines/cad2014.pdf) CPG  Changed price according to DPRI (0.91) + 30% markup (1.083) | 1.0 | 1 | 365 | 365 | 1.083 | 394.2 |
| Oxygen, 1000 liters, primarily with oxygen cylinders | 100 | 4 L/min (5.76 cubic maters), for 24 hours | 0.0 | 1,400 | 1 | 0 | 315 | 864.66 |
| IV line | 100 |  | 1.0 | 1 | 2 | 2 | 20 | 40 |
| Saline solution | 100 |  | 1.0 | 2 | 14 | 28 | 65 | 1,820 |
| MEDICAL PERSONNEL | | | | | | | | |
| Staff type | % | Note | Minutes | | # of days/visits | | | Total |
| Generalists | 100 | Default | 20 | | 1 | | | 20 |
| Nurse | 100 |  | 10 | | 4 | | | 40 |
| OUTPATIENT VISITS AND INPATIENT DAYS | | | | | | | | |
| Type | % | Note | Units per case | | | | | Total |
| Outpatient visit | 100 | Default | 5 | | | | | 5 |
| Inpatient days | 100 |  | 2 | | | | | 2 |

Table 3.9. Treatment inputs for **Treatment of cases with established ischaemic heart disease (IHD) and post MI** in GCEA

| **Intervention Name: Treatment of cases with established ischaemic heart disease (IHD) and post MI** | | | | | | | | |
| --- | --- | --- | --- | --- | --- | --- | --- | --- |
| DRUGS AND SUPPLIES | | | | | | | | |
| Drug/supply | % rec asp of treat | Note | # units | Times per day | Days per case | Units per case | Unit cost (PHP) 2015 | Cost per ave case (PHP) 2015 |
| Drugs and supplies required per client | | | | | | | | |
| Acetyl salysilic acid (aspirin), tablet, 80 mg | 100 | See comment in Table 3.8 | 1.0 | 1 | 365 | 365 | 1.083 | 394.2 |
| Enalapril, tablet, 5 mg | 100 | See comment in Table 3.4 | 1.0 | 1 | 365 | 365 | 5.005 | 1,826.83 |
| Atenolol, tablet,s 50 mg | 100 | See comment in Table 3.4 | 1 | 1 | 365 | 365 | 1.638 | 597.87 |
| Simvastatin, 15 mg | 100 | See comment in Table 3.3 | 1 | 1 | 365 | 365 | 2.405 | 877.83 |
| Prednisolone, tablet, 5 mg |  | Removed because COPD oral medication not explicitly indicated in Philippine PEN guidelines |  |  |  |  |  |  |
| Laboratory tests per client | | | | | | | | |
| Blood glucose level test | 87.0 | Default kept | 1.0 | 1 | 2 | 2 | 140 | 243.60 |
| Follow-up for those with diabetes | | | | | | | | |
| Blood glucose level test | 13.0 |  | 1.0 | 1 | 20 | 20 | 140 | 364.00 |
| Cholesterol test | 100.0 |  | 1.0 | 1 | 2 | 2 | 91 | 182 |
| Urine analysis | 100.0 |  | 1.0 | 1 | 2 | 2 | 75 | 150 |
| MEDICAL PERSONNEL | | | | | | | | |
| Staff type | % | Note | Minutes | | # of days/visits | | | Total |
| Generalists | 100 |  | 5 | | 6 | | | 30 |
| Nurse | 13 |  | 20 | | 4 | | | 10.40 |
| OUTPATIENT VISITS AND INPATIENT DAYS | | | | | | | | |
| Type | % | Note | Units per case | | | | | Total |
| Standard treatment | | | | | | | | |
| Outpatient visit | 100 | Default | 6 | | | | | 6 |
| Additional follow-up for those with diabetes | | | | | | | | |
| Outpatient visit | 13.0 | Default | 4 | | | | | 0.5 |

Table 3.10. Treatment inputs for **Treatment for those with established cerebrovascular disease and post stroke** in GCEA

| **Intervention Name: Treatment for those with established cerebrovascular disease and post stroke** | | | | | | | | |
| --- | --- | --- | --- | --- | --- | --- | --- | --- |
| DRUGS AND SUPPLIES | | | | | | | | |
| Drug/supply | % rec asp of treat | Note | # units | Times per day | Days per case | Units per case | Unit cost (PHP) 2015 | Cost per ave case (PHP) 2015 |
| Drugs and supplies required per client | | | | | | | | |
| Acetyl salysilic acid (aspirin), tablet, 80 mg | 100 | See comment in Table 3.8 | 1.0 | 1 | 365 | 365 | 1.083 | 394.2 |
| Enalapril, tablet, 5 mg | 100 | See comment in Table 3.4 | 1.0 | 1 | 365 | 365 | 5.005 | 1,826.83 |
| Atenolol, tablets, 50 mg | 100 | See comment in Table 3.4 | 1 | 1 | 365 | 365 | 1.638 | 597.87 |
| Simvastatin, 15 mg | 100 | See comment in Table 3.3 | 1 | 1 | 365 | 365 | 2.405 | 877.83 |
| Prednisolone, tablet, 5 mg |  | Removed because COPD oral medication not explicitly indicated in Philippine PEN guidelines |  |  |  |  |  |  |
| Follow-up for those with diabetes | | | | | | | | |
| Blood glucose level test | 13.0 |  | 1.0 | 1 | 20 | 20 | 140 | 364 |
| Laboratory tests per client | | | | | | | | |
| Blood glucose level test | 87.0 |  | 1.0 | 1 | 2 | 2 | 140 | 243.6 |
| Cholesterol test | 100.0 |  | 1.0 | 1 | 2 | 2 | 91 | 182 |
| Urine analysis | 100.0 |  | 1.0 | 1 | 2 | 2 | 75 | 150 |
| MEDICAL PERSONNEL | | | | | | | | |
| Staff type | % | Note | Minutes | | # of days/visits | | | Total |
| Standard treatment | | | | | | | | |
| Generalists | 100 |  | 5 | | 6 | | | 30 |
| Follow-up for those with diabetes | | | | | | | | |
| Nurse | 13 |  | 20 | | 4 | | | 10.40 |
| Physiotherapy | | | | | | | | |
| Nurse | 20 |  | 20 | | 4 | | | 16 |
| OUTPATIENT VISITS AND INPATIENT DAYS | | | | | | | | |
| Type | % | Note | Units per case | | | | | Total |
| Standard treatment | | | | | | | | |
| Outpatient visit | 100 | Default | 2 | | | | | 2 |
| Inpatient days | 100 |  | 6 | | | | | 6 |
| Follow-up for those with diabetes | | | | | | | | |
| Outpatient visit | 13.0 | Default | 4 | | | | | 0.5 |
| Physiotherapy | | | | | | | | |
| Outpatient visit | 20 | Default | 4 | | | | | 0.8 |

Table 3.11. Treatment inputs for **Treatment of cases with rheumatic heart disease (with benzathine penicillin)** in GCEA

| **Intervention Name: Treatment of cases with rheumatic heart disease (with benzathine penicillin)** | | | | | | | | |
| --- | --- | --- | --- | --- | --- | --- | --- | --- |
| DRUGS AND SUPPLIES | | | | | | | | |
| Drug/supply | % rec asp of treat | Note | # units | Times per day | Days per case | Units per case | Unit cost (PHP) 2015 | Cost per ave case (PHP) 2015 |
| Penicillin G Benzathine, 1.2 million IU | 100 | Changed drug and dosage because this is the recommended dose according to Philippine Society of Pediatric Cardiology (PSPC) [RF/RHD CPG](http://www.thefilipinodoctor.com/cpm_pdf/CPM3rd%20Rheumatic%20fever.pdf)  Recommended dose once a month, delivered intravenously  Changed price according to DPRI (41.98) + 30% markup (54.57) | 1 | 1 | 12 | 12 | 54.57 | 654.84 |
| MEDICAL PERSONNEL | | | | | | | | |
| Staff type | % | Note | Minutes | | # of days/visits | | | Total |
| Generalists |  | Removed, not required anymore - a nurse can deliver the shot |  | |  | | |  |
| Nurse | 100 | Reduced time to 5 minutes | 5 | | 12 | | | 60 |
| OUTPATIENT VISITS AND INPATIENT DAYS | | | | | | | | |
| Type | % | Note | Units per case | | | | | Total |
| Outpatient visit | 100 | Changed to reflect number of monthly visits to nurse | 12 | | | | | 12 |
| Inpatient days |  | Removed, not required |  | | | | |  |

### Delivery Channels

Table 3.12. Percent of interventions delivered at different levels

|  | Community | Outreach | Clinic | Hospital | Notes |
| --- | --- | --- | --- | --- | --- |
| Screening for risk of CVD/diabetes |  |  | 100 | 0 | Assumed to be default PEN guidelines |
| Follow-up care for those at low risk of CVD/diabetes (absolute risk: 10-20%) |  |  | 100 | 0 |  |
| Treatment for those with very high cholesterol but low absolute risk of CVD/diabetes (<20%) |  |  | 100 | 0 |  |
| Treatment for those with high blood pressure but low absolute risk of CVD/diabetes (<20%) |  |  | 100 | 0 |  |
| Treatment for those with absolute risk of CVD/diabetes (20-30%) |  |  | 100 | 0 |  |
| Treatment for those with high absolute risk of CVD/diabetes (>30%) |  |  | 50 | 50 |  |
| Treatment of new cases of acute myocardial infarction (AMI) with aspirin |  |  |  | 100 |  |
| Treatment of cases with established ischaemeic heart disease (IHC) and post MI |  |  |  | 100 |  |
| Treatment for those with established cerebrovascular disease and post stroke |  |  |  | 100 |  |
| Treatment of cases with rheumatic heart disease (with benzathine penicillin) |  |  | 50 | 50 |  |
