# Supplementary material for: Reflections on the use of the World Health Organization’s (WHO) OneHealth Tool: Implications for health planning in low and middle income countries (LMICs)
Source: F1000Res. 2018 Mar 5;7:157. Originally published 2018 Feb 7. [Version 2] doi: 10.12688/f1000research.13824.2 (PMC5832917; doi:10.12688/f1000research.13824.2)
Supplement: Peer review history

# Peer review: Referee response for version 1

**DOI:** 10.5256/f1000research.15027.r30629

**Published:** 2018-03-01

**Authors:** Lotte M.G. Steuten

**Affiliations:** 1 Hutchinson Institute for Cancer Outcomes Research, Fred Hutchinson Cancer Research Center, Seattle, WA, USA

The authors have described their reflections on the experience with Generalized Cost Effectiveness Analysis (GCEA) using the WHO One Health Tool (OHT), to aid other Low-Middle Income Countries (LMICs) looking to scale up their priority setting efforts.

 They clearly describe the rationale and aim of using this tool to support redefining the benefits package of the national insurer of the Philippines. Most importantly, they identify key limitations on various levels: 1) limitations of the OHT itself; 2) limitations in availability of models and data specific to the Philippine context; and 3) the unmet need to establish a single, harmonized priority setting process for the health sector that would enable multiple stakeholders (in this case the Department of Health and the Philippine Health Insurance Corporation) to make better policy decisions based on the questions they are currently faced with. Based on these limitations and their experiences the authors provide general recommendations that are relevant to the Philippines and to other LMICs.

 The paper is valuable and sound, given that its aims are quite humbly to share reflections on the use of the OHT for a priority setting exercise. However, these reflections –however well described and sensible- should not be read as (and are not intended to be) the result of a systematic evaluation of experiences based on all stakeholders inputs. A degree of subjectivity should be acknowledged and the paper would have benefit from more specific or detailed suggestions to improve the priority setting exercise and experience, from the perspective of the different participants. That said, this paper is a valuable contribution to the literature and more examples such as these should be welcomed and shared to drive the HTA field forward, globally as well as locally.

I have read this submission. I believe that I have an appropriate level of expertise to confirm that it is of an acceptable scientific standard.

# Peer review: Referee response for version 1

**DOI:** 10.5256/f1000research.15027.r30630

**Published:** 2018-02-08

**Authors:** Kalipso Chalkidou

**Affiliations:** 1 Global Health and Development Team, Imperial College London, London, UK

This is a valuable and original contribution describing the analytical methods, data sources and overall policy approach to reviewing and attempting to adjust the health benefits package in the Philippines (based on a selection of mostly NCD interventions) using WHO’s OneHealth tool. A number of middle income countries are currently in a similar position, *en route* to Universal Healthcare Coverage and making a systematic attempt at revising their Benefits Package as part of the national or social health insurance funds and not many resources exist on how such a process can be carried out (e.g. see https://www.cgdev.org/publication/whats-in-whats-out-designing-benefits-universal-health-coverage for the Centre for Global Development guide to designing benefits packages).

 The authors succinctly summarise the challenges they faced including the limited breadth of the OneHealth models available to cover the whole spectrum of diseases the country faces. They also highlight the problematic assumption of the “null comparator”. The latter is a core element of the WHO approach to cost effectiveness analysis and makes structuring the decision problem in a way that reflects the reality policy makers are faced with, where the option of doing nothing is neither realistic nor ethical, impossible.

 The authors also flag up as major weaknesses the lack of national guidelines on the methods and processes for revising the package. Again there is limited global guidance as to best practice in the methods (for one example see the iDSI Reference Case to economic evaluation here http://www.idsihealth.org/resource-items/idsi-reference-case-for-economic-evaluation/) and processes for managing such a methodologically and politically challenging exercise.

 We need more such examples to be publicised and for a dialogue to start within and between countries as to how best to support policy makers achieve UHC in a fair and sustainable way. The authors may not have the answers but pose important questions for driving discussion as well as research.

I have read this submission. I believe that I have an appropriate level of expertise to confirm that it is of an acceptable scientific standard.
